# Transcriptomic Analysis of the *Campylobacter jejuni* Response to T4-Like Phage NCTC 12673 Infection

**DOI:** 10.3390/v10060332

**Published:** 2018-06-16

**Authors:** Jessica C. Sacher, Annika Flint, James Butcher, Bob Blasdel, Hayley M. Reynolds, Rob Lavigne, Alain Stintzi, Christine M. Szymanski

**Affiliations:** 1 Department of Biological Sciences, University of Alberta, Edmonton, AB T6G 2E9, Canada; cszymans@uga.edu; 2 Ottawa Institute of Systems Biology, Department of Biochemistry, Microbiology and Immunology, University of Ottawa, Ottawa, ON K1H 8M5, Canada; aflin053@gmail.com (A.F.); jbutcher1@gmail.com (J.B.); astintzi@uottawa.ca (A.S.); 3 Laboratory of Gene Technology, Department of Biosystems, KU Leuven, Leuven 3001, Belgium; blasdelb@gmail.com (B.B.); rob.lavigne@kuleuven.be (R.L.); 4 Department of Microbiology and Complex Carbohydrate Research Center, University of Georgia, Athens, GA 30602, USA; hayley.reynolds25@uga.edu

**Keywords:** *Campylobacter jejuni*, bacteriophage, NCTC 12673, transcriptome, phage defense

## Abstract

*Campylobacter jejuni* is a frequent foodborne pathogen of humans. As *C. jejuni* infections commonly arise from contaminated poultry, phage treatments have been proposed to reduce the *C. jejuni* load on farms to prevent human infections. While a prior report documented the transcriptome of *C. jejuni* phages during the carrier state life cycle, transcriptomic analysis of a lytic *C. jejuni* phage infection has not been reported. We used RNA-sequencing to profile the infection of *C. jejuni* NCTC 11168 by the lytic T4-like myovirus NCTC 12673. Interestingly, we found that the most highly upregulated host genes upon infection make up an uncharacterized operon (*cj0423–cj0425*), which includes genes with similarity to T4 superinfection exclusion and antitoxin genes. Other significantly upregulated genes include those involved in oxidative stress defense and the *Campylobacter* multidrug efflux pump (CmeABC). We found that phage infectivity is altered by mutagenesis of the oxidative stress defense genes catalase (*katA*), alkyl-hydroxyperoxidase (*ahpC*), and superoxide dismutase (*sodB*), and by mutagenesis of the efflux pump genes *cmeA* and *cmeB*. This suggests a role for these gene products in phage infection. Together, our results shed light on the phage-host dynamics of an important foodborne pathogen during lytic infection by a T4-like phage.

## 1. Introduction

Bacteriophages (phages), the viruses that infect bacteria, represent a diverse class of natural bacterial predators that have shaped bacterial evolution for an estimated three billion years. Studying how phages interact with their hosts can highlight important facets of bacterial biology and inspire new ways of controlling bacterial pathogens. As antibiotic resistant infections now threaten to cause more deaths per year than cancer by 2050 [1], there is a great need to explore alternative antimicrobial therapies. Phages represent a viable antibiotic alternative, both in human healthcare settings and in agriculture [2,3,4]. To ensure the safety and efficacy of employing phages in these settings, only well-characterized phages should be used.

Since the discovery of phages more than a century ago, phage-host characterization has progressed through many stages, from examining plaque morphology, to molecular genetics using model phage-host systems, to classifying phages by transmission electron microscopy, to genome sequencing and annotation. Each of these techniques is useful, and the information gained from each strategy continues to feed information into the still-accelerating collective understanding of phage biology. However, these techniques provide limited information about the host response to infection or the extent to which a phage manipulates host metabolism during infection. As a result, many questions regarding phage infection mechanisms and the host factors that help or hinder these processes remain to be answered for most phages. However, recent advances in whole-genome and whole-transcriptome sequencing now accommodate a much more detailed analysis of both phage and host gene expression profiles during an infection [5]. Within the last few years, whole-transcriptome sequencing methods such as RNA-sequencing (RNA-seq) have been applied to several phage-host pairs, and are rapidly providing a wealth of information on phage infection and assembly programs, host responses to phages, and the mechanisms these phages use to counter these responses [6,7,8,9,10,11,12,13,14,15,16].

*C. jejuni* is a frequent foodborne pathogen of humans. Strain NCTC 11168 was the first to be genome-sequenced [17,18], and remains one of the best characterized of the species. As *C. jejuni* infections tend to arise from handling and consuming contaminated poultry [19,20], phages have been considered as a method of reducing the *C. jejuni* load on farms to prevent human infections [4,21,22,23]. While a prior report described the transcriptomes of two *C. jejuni* phages, CP30A and CP8, during carrier-state association with their host, *C. jejuni* PT14 [11], transcriptomic analysis of a lytic *C. jejuni* phage infection has not yet been reported.

NCTC 12673 phage is a 135-kB T4-like myovirus that was isolated from poultry [24]. Previously, we sequenced the NCTC 12673 genome and identified 172 ORFs and three tRNA genes [25]. Interestingly, NCTC 12673 is more similar to T4-related cyanobacteria specific myoviruses than to other phages targeting proteobacteria [25]. NCTC 12673 is a group III *Campylobacter* phage, which is a member of the *Cp8virus* genus of the *Eucampyvirinae* subfamily of the *Myoviridae* [26]. Members of this group tend to require capsular polysaccharide (CPS) for infection [27,28], and indeed, host range analysis revealed that NCTC 12673 is CPS-dependent [28]. No CPS-specific receptor binding protein (RBP) has yet been identified for this phage, although it was previously thought that Gp047 (formerly Gp48) served this function. However, extensive characterization of Gp047 has shown that this protein is a flagellar glycan-specific protein [29,30]. NCTC 12673 encodes its own DNA polymerase, but no RNA polymerase has so far been annotated [25].

In this report, we used RNA-seq to profile phage-host interactions between phage NCTC 12673 and *C. jejuni* NCTC 11168 during infection. To better understand the impact of host gene expression changes upon phage infection, we used targeted mutagenesis to confirm the role of selected upregulated host pathways on phage infectivity. We identified possible roles for oxidative stress defense proteins and for the CmeABC multi-drug efflux pump in phage infectivity. We also found that the uncharacterized *C. jejuni* operon *cj0423–cj0425* made up the most highly upregulated genes upon infection. Together, our results highlight new aspects of *C. jejuni* phage-host interactions and contribute to the understanding of phage infection dynamics among the T4-like phages.

## 2. Materials and Methods

### 2.1. Bacterial Growth Conditions

*C. jejuni* NCTC 11168 (MP21) [31] and its isogenic deletion mutants were grown on 1.5% NZCYM (Research Products International, Mt Prospect, IL, USA) agar plates, supplemented with 50 µg/mL kanamycin or 12–25 µg/mL chloramphenicol where needed, at 37 °C under microaerobic conditions (85% N_2_, 10% CO_2_, 5% O_2_). *Escherichia coli* strains were grown on LB agar, supplemented with 50 µg/mL kanamycin or 25 µg/mL chloramphenicol where needed. The list of bacterial strains and phages used in this study is given in Table 1.

### 2.2. Phage Propagation, Titration and Concentration

Phage NCTC 12673 and its propagating strain, *C. jejuni* 12661 [32], were obtained from the National Collection of Type Cultures (NCTC; Salisbury, United Kingdom). Phage propagation and titration were performed following the methods described in [33], except that 4-h pre-growth of cultures was done with NZCYM broth instead of cation-adjusted brain heart infusion (cBHI) broth. To increase phage titres prior to RNA-sequencing experiments, 0.22-μm-filtered phage lysates were ultracentrifuged at 207,870 × *g* in a swinging bucket rotor (SW-28 Ti, Beckman Coulter, Indianapolis, IN, USA) for 1.5 h at 4 °C. Pellets from 25-mL volumes of lysate were resuspended in 1 mL SM buffer containing gelatin (Cold Spring Harbor Laboratory, Laurel Hollow, NY, USA) [33], pooled, and titred on *C. jejuni* NCTC 11168 cells.

### 2.3. Total RNA Extraction

Cells were harvested from overnight NZCYM plate cultures, pelleted, washed once in NZCYM broth and set to an OD_600_ of 0.05 (2 × 10^8^ colony forming units per mL (CFU/mL)) in 20 mL NZCYM broth in 125-mL Erlenmeyer flasks, each containing a 1-inch sterile magnetic stir-bar. Cells were grown under microaerobic conditions and magnetically stirred at 200 rpm. After 4.5 h incubation (mid-log phase, cells had achieved approximately 5.65 +/− 0.79 × 10^8^ CFU/mL), 6.5 mL NCTC 12673 phage (2.33 × 10^9^ plaque forming units per mL (PFU/mL) in SM buffer) was added to each of the three flasks, giving a multiplicity of infection (MOI) of approximately 1.37 +/− 0.25. As negative controls, 6.5 mL SM buffer was added to each of the three other flasks. At t = 30 min, 1 h, and 2 h, one phage-containing and one buffer-containing flask was removed from the incubator, and the entire contents of each flask were transferred to a pre-prepared tube containing 2.6 mL (0.1 volume) ice cold 10% buffered phenol in 100% ethanol to stabilize RNA, followed by immediate mixing and storage on ice until all samples were collected [34]. RNA was extracted from each sample using a hot phenol method [34]. RNA samples were sequentially DNAse-treated (37 °C for 30 min) using RNAse-free DNAse I (Epicentre, Madison, WI, USA) and cleaned using the Zymo RNA Clean & Concentrator (Zymo Research, Irvine, CA, USA). PCR was used to confirm the absence of residual DNA. Total RNA quality was assessed using an Agilent Bioanalyzer (Agilent Technologies, Santa Clara, CA, USA) and RNA was stored at −80 °C until further use. Samples were extracted in biological triplicate.

### 2.4. RNA Sequencing

Total mRNA libraries from all replicates were generated. Samples were depleted of rRNA using the RiboZero Bacterial kit (Illumina, San Diego, CA, USA) according to the manufacturer’s instructions. Successful rRNA depletion was confirmed using the Agilent Bioanalyzer RNA 6000 Pico Kit (Agilent Technologies, Santa Clara, CA, USA). Strand-specific barcoded sequencing libraries were constructed using the Ion Total RNA-seq kit (Thermo Fisher Scientific, Waltham, MA, USA). Libraries were quality-checked and quantified using the Bioanalyzer High Sensitivity DNA kit (Agilent Technologies, Santa Clara, CA, USA) and pooled together in equimolar amounts. The pooled libraries were templated using the Ion PI Hi-Q kit (Thermo Fisher Scientific, Waltham, MA, USA) and sequenced on an Ion Torrent Proton using the Ion PI Hi-Q sequencing 200 kit (Thermo Fisher Scientific, Waltham, MA, USA) on a single Proton V2 chip.

The raw sequencing reads were demultiplexed by the Ion Torrent suite software (version 5.2.2., Thermo Fisher Scientific, Waltham, MA, USA) and sequentially mapped to the host (NCTC 11168) and phage (NCTC 12673) genomes using the Spliced Transcripts Alignment to a Reference (STAR) method [35] (Table S1). Reads aligning to coding regions were counted using HT-seq using the default settings [36]. The raw demultiplexed sequencing reads have been deposited at the NCBI Sequence Read Archive (SRA) under accession number PRJNA454099. DESeq2 was used to identify differentially expressed transcripts over time and between different time points. Genes with a fold change +/− 1.5 and false discovery rate (FDR)-corrected *p*-value < 0.05 were considered differentially expressed. Host and phage gene expression was analyzed separately. We also conducted gene set enrichment analysis (GSEA) on *C. jejuni* Kyoto Encyclopedia of Genes and Genomes (KEGG) pathways [37] using the generally applicable gene set enrichment (GAGE) method [38] with a minimum FDR cutoff of <0.1 [35].

**Figure 1:**
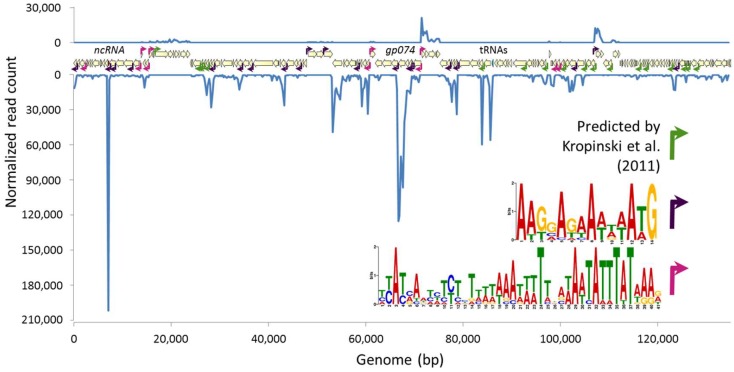
RNA read counts across the NCTC 12673 phage genome aligning to the Watson (top) and Crick (bottom) strands. Previously predicted promoters [25] are indicated by green arrows, while two newly predicted promoter motifs are indicated with fuchsia and purple arrows. Coding sequences are indicated with beige arrows, while the tRNA region is indicated with a blue arrow and a newly identified ncRNA is highlighted with a red bar.

Figure 1 was assembled by summarizing phage reads from all infected samples into count tables of total gene reads that align to each 250-bp section of the genome independently for each strand. These counts were then graphed independently for each strand. Putative promoters were predicted by systematically identifying clear transcription start sites indicated by RNA-Seq data, and searching for motifs on the relevant strand in the region 50 bp upstream and 150 bp downstream using Multiple Em for Motif Elicitation (MEME v4.12.0, [39]). The phage genome was then scanned for these motifs using Find Individual Motif Occurrences (FIMO), and additional examples were annotated (FIMO v4.12.0, [40]).

### 2.5. Efficiency of Plating (EOP) Assays

Efficiency of plating (EOP) assays were conducted by spotting serial dilutions of NCTC 12673 phage onto different strains and determining the proportion of plaque forming units formed on mutant strains compared to the corresponding wild type strain. Briefly, overnight bacterial cultures were harvested in NZCYM broth and set to an OD_600_ of 0.35. A 5-mL aliquot of this suspension was transferred to a standard sized empty Petri dish and incubated at 37 °C without shaking for 4 h under microaerobic conditions. The suspension was then set to an OD_600_ of 0.5, and 200 µL of this was mixed with 5 mL sterile 0.6% molten NZCYM agar at 45 °C. This suspension was poured onto the surface of a pre-warmed NZCYM plate containing 1.5% agar. Plates were allowed to solidify for 15 min and then 10 µL of serial dilutions of a phage suspension (starting at 10^7^ PFU/mL) was spotted onto the agar surface and allowed to completely soak into the agar (15 min) before inverting the plate and incubating it at 37 °C under microaerobic conditions. Plaques were counted after 18–24 h and converted to PFU/mL by multiplying countable numbers by the total dilution factor. This method is stored on Protocols.io (http://dx.doi.org/10.17504/protocols.io.mahc2b6).

## 3. Results

### 3.1. NCTC 12673 Phage Infection Resulted in Clear Differences in Overall *C. jejuni* Gene Transcription

To better understand *Campylobacter* phage-host dynamics during lytic infection, we performed RNA-seq on *C. jejuni* NCTC 11168 cells at three time points (30, 60, and 120 min) post NCTC 12673 phage infection. These time points were selected to capture cellular responses to phages prior to lysis of the cultures [44]. We grew 20-mL *C. jejuni* cultures in NZCYM broth until the mid-log phase before infecting them with phages at a multiplicity of infection (MOI) of approximately 1. We then sequenced total mRNA from infected and mock-infected samples to identify differentially expressed host genes at each time point. Importantly, the use of a relatively low MOI allows changes in gene expression of both phage-infected and uninfected *C. jejuni* cells to be examined concurrently, as RNA from both populations is sequenced. As others have observed that signals transmitted from phage-infected cells to uninfected sister cells can have important impacts on their physiology [14,45,46], we were also interested in the responses of the uninfected population during infection. Of note, this type of analysis does not allow the attribution of gene expression differences to one population or the other, but instead surveys the pooled responses of both infected and uninfected cells. Infection with the NCTC 12673 phage resulted in clear differences in the overall gene transcription between the groups. Notably, the infected and uninfected controls showed clear separation using principal component analysis; however, we did not note any clear trends for the transcriptomes to separate them by sampling time (Figure S1).

### 3.2. Phage Transcriptional Analysis Validates in Silico Predictions and Suggests Two New Promoter Motifs

Although information about temporal phage gene regulation can only be determined for synchronously infected cells, considerable information about the genetic structure underlying phage gene expression, including the identification of transcription start and stop sites, promoter sequences, and non-coding RNA species, can be gleaned from a study of this design. We analyzed whether read counts across the phage genome mapped according to predicted transcription start and stop sites. We found that the host promoters and Rho-independent terminators predicted by [25] could be validated by RNA-Seq data, though most transcription appears to be driven by one of two novel A/T rich predicted promoter motifs (Figure 1).

The NCTC 12673 phage is predicted to encode 171 ORFs, and we detected transcripts from 166 ORFs [25]. The five ORFs without any assigned reads correspond to *gp013*, *gp054, gp071, gp081*, and *gp124,* which are predicted to encode pseudogenes. Of the 166 transcribed genes detected, only 62 (37%) have predicted functions. No statistically significant differences in phage gene expression were identified among different time points post infection. The most densely transcribed feature in the phage genome was a previously unannotated 158-bp non-coding RNA between *gp012* and *gp013* (Figure S2)*.* However, the major capsid protein-encoding gene, *gp074,* was the most highly expressed phage coding sequence (Table S2). Average reads per kilobase of transcript per million mapped reads (RPKM) values for genes encoding products detected in the structural proteome for this phage [25] were greater than those corresponding to genes not detected in the proteome (*p* < 10^−9^ by Student’s *t*-test). Although the NCTC 12673 phage encodes several homologues to structural protein-encoding genes commonly shared among T4-like phages, seven of the top ten most highly expressed NCTC 12673 protein-encoding genes lack predicted functions.

### 3.3. Phage Infection Induces Widespread Induction of Host Genes and Downregulation of Energy Metabolism Pathways

To identify cellular processes important for the infection program of phage NCTC 12673 and to identify host responses to phage infection, we analyzed differentially expressed host genes and pathways during infection at an MOI of about 1. We observed an increasing number of differentially expressed host genes over time in response to phage infection, with many more genes upregulated than downregulated at all time points (Table S3). At 30 min p.i., we observed upregulation of 3.7% and downregulation of 0.06% of host genes; at 60 min, we observed upregulation of 1.9% and downregulation of 0.5% of host genes; and at 120 min, we observed upregulation of 12.7% and downregulation of 4.7% of host genes. However, as the number of infected cells at any given time under our experimental conditions may be very low, these proportions are not necessarily representative of the changes in host transcription occurring on a single-cell level upon infection.

To gain a broad view of which host metabolic pathways were differentially expressed upon phage infection, we analyzed the enrichment of KEGG categories among upregulated and downregulated genes. Among upregulated genes, only the KEGG ribosome pathway (cje03010) was statistically enriched during infection (FDR-adjusted *p* = 0.003, 120 min). However, among downregulated genes, we observed the statistically significant enrichment of many KEGG pathways pertaining to energy metabolism, such as pyruvate, propanoate, and butanoate metabolism pathways and the TCA cycle (Table S4). This suggests that energy metabolism is decreased upon NCTC 12673 phage infection, but that translation may increase upon infection.

### 3.4. Genes Involved in DNA, RNA, Amino Acid, and Protein Synthesis are Upregulated in Infected Cultures

As would be expected to be important for phage replication based on studies of T4 and other T4-like phages [9,47,48,49], we identified several genes involved in DNA synthesis, replication, and repair, as well as transcription and translation, to be differentially expressed upon NCTC 12673 phage infection of *C. jejuni* cultures (Table S5). When genes in these categories were differentially expressed, they were nearly always upregulated, and statistically significant differences were predominantly only observed at the last time point.

Although the phage itself encodes its own ribonucleotide reductases (*gp162* and *gp163*), the two host ribonucleotide reductase subunits *nrdA* and *nrdB* were upregulated at 120 min p.i (+1.9-fold and +1.6-fold, respectively). The phage also encodes its own DNA polymerase (*gp101*), but we also observed that the host DNA polymerase III delta prime subunit (*cj0584*) was upregulated by 1.9-fold at 120 min p.i. The purine biosynthesis genes *purBHLE* were also upregulated at 120 min p.i. (+2-fold each). Of note, *purE* was downregulated twofold at 60 min p.i., yet was still upregulated similarly to the other *pur* genes at 120 min. In terms of genes associated with DNA modification and repair, the EcoRI-like adenine-specific methylase *cj0208*, the G:T/U mismatch glycosylase *cj1254*, and the exoDNAse VII *xseA* were upregulated 2.9-, 2.6-, and 1.9-fold, respectively, at 120 min. As the NCTC 12673 phage does not encode its own RNA polymerase, similar to T4 phage, we expected to observe the upregulation of host RNA polymerase genes upon phage infection, and indeed the RNA polymerase subunits *rpoABC* were upregulated 1.9-, 2.0-, and 1.6-fold, respectively, at 120 min. As anticipated during a lytic phage infection, and in line with our KEGG pathway analysis results, we also observed the upregulation of many ribosomal protein-encoding genes. We also observed several tRNA modification genes to be upregulated, as well as one, seryl-tRNA synthetase (−1.6-fold at 120 min p.i.), which was downregulated.

Several genes involved in amino acid metabolism were upregulated by 120 min p.i., including arginine biosynthesis genes *argBCD*, which were all upregulated by 2.7–3.3 fold. Additionally, the branched chain amino acid transport genes *livFHJ* were upregulated 1.4–1.8 fold, and the leucine biosynthesis genes *leuACD* were each upregulated by two-fold. Not all differentially expressed genes involved in amino acid biosynthesis were upregulated. The aspartate aminotransferase gene *aspB*, which is essential for growth on glutamate [50], and the aspartate/glutamate transport genes *cj0919c*, *cj0920c*, and *peb1A*, were all downregulated 1.6- to 2.5-fold by 120 min p.i., as were the proline and serine biosynthesis/transport genes *proA*, *putP*, and *sdaAC*, which were downregulated approximately two-fold at 120 min p.i.

### 3.5. Canonical Phage Defense Systems are not Differentially Expressed Upon Phage Infection

We have previously observed that phage NCTC 12673 infects *C. jejuni* NCTC 11168 to a lesser extent (one to two orders of magnitude less efficiently) than it infects its propagating host, *C. jejuni* NCTC 12661 [51]. Therefore, we hypothesized that phage defense systems expressed by NCTC 11168 cells might act to reduce NCTC 12673 phage infection efficiency. NCTC 11168 is known to encode a type II-C CRISPR-Cas system and type I and type II restriction modification systems [52,53,54,55]. However, the only gene belonging to either category that was upregulated upon infection was *cj0140*, which is predicted to encode the McrC component of a McrBC 5-methylcytosine restriction system (+1.75-fold, 120 min p.i.) (Table S5). The McrBC system is known to play a role in *E. coli*-mediated degradation of modified cytosine-containing DNA, and is thought to have led to T4 phage evolution of glycosylated hydroxymethylcytosine in its DNA [47]. Whether this system plays a role in *C. jejuni* phage defense remains to be determined, but the fact that McrC is upregulated upon NCTC 12673 infection is intriguing. No other restriction endonuclease system genes and no CRISPR-Cas genes were upregulated, suggesting that these systems are not induced by NCTC 12673 infection.

Another common method of phage defense is through the alteration of surface receptors. NCTC 12673 phage is CPS-specific [28]. Of note, two genes annotated as glycosyltransferases, *cj1434c* and *cj1442c*, which are both found in the *C. jejuni* NCTC 11168 CPS locus, were upregulated two- to three-fold at 60 and 120 min post infection (Table S5). We also noticed that two other CPS-associated genes, *cj1420c* and *cj1422c*, which encode a putative methyltransferase and putative O-methylphosphoramidate (MeOPN) transferase, respectively [56], were each upregulated approximately two-fold at 120 min upon NCTC 12673 infection, though *cj1420* was also downregulated 1.7-fold at 60 min.

### 3.6. Host Operons *cmeABC*, *chuABCD* and *cj0423–cj0425* are Among the Most Highly Upregulated upon Phage Infection

**Figure 2:**
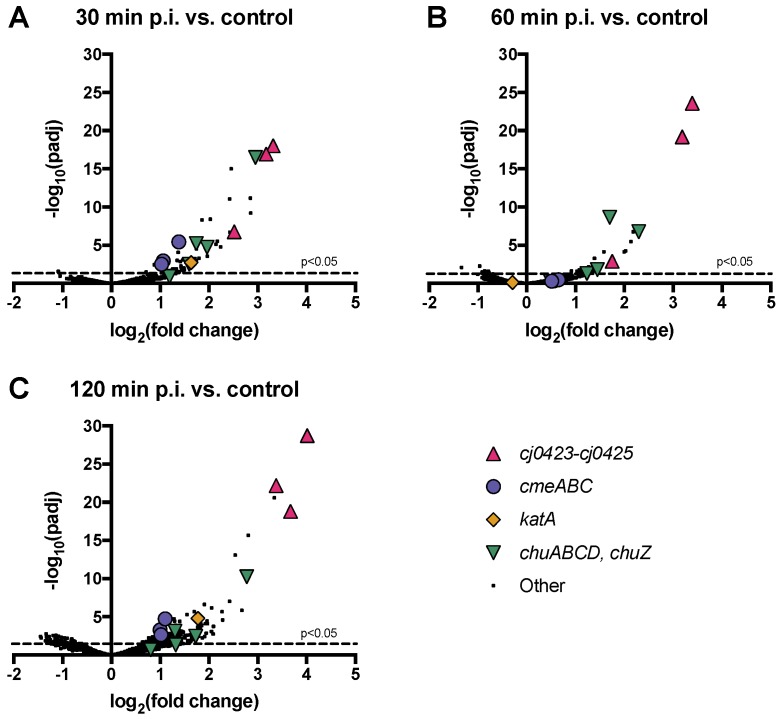
Many *C. jejuni* NCTC 11168 genes are upregulated in response to NCTC 12673 phage infection, while relatively few are downregulated. Volcano plots show all transcribed *C. jejuni* NCTC 11168 genes at (**A**) 30, (**B**) 60, and (**C**) 120 min post infection by NCTC 12673 phage compared to mock-infected controls at each time point. The negative log of the false discovery rate-adjusted *P-*value vs. the log_2_ fold change between the conditions indicated is plotted for each gene. Selected highly significantly differentially expressed genes are represented by different colours and symbols according to their predicted or known function.

We sought to determine whether any upregulated host pathways affected phage infection, either as stress or defense mechanisms upregulated by infected or surrounding cells, or as phage-induced pathways that might facilitate a cellular environment more favourable to infection. To identify the most highly phage-responsive genes with the strongest associated statistical significance, we plotted significance versus fold change for all host genes (Figure 2). We identified three operons that were highly expressed during host infection: *cmeABC, chuABCD* (plus *chuZ*, which is expressed from a different promoter but located adjacently to *chuABCD*), and the uncharacterized operon *cj0423–cj0425* (Figure 2, Table 2). In all cases, all genes making up the operon displayed high fold change and high significance values at one or more time points.

#### 3.6.1. The *C. jejuni* CmeABC Multi-Drug Efflux Pump May Function in Phage Defense

**Figure 3:**
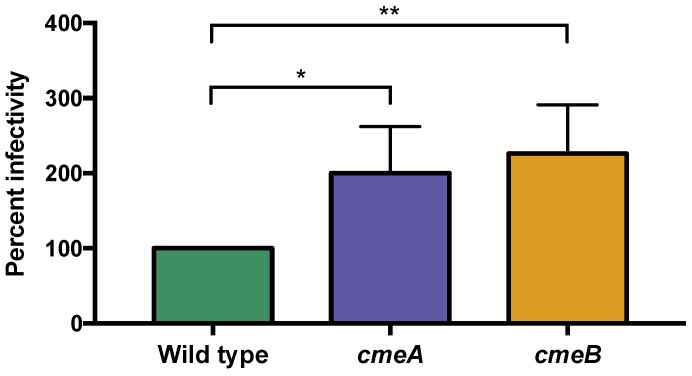
NCTC 12673 phage efficiency of plating is greater on a *cmeA* and *cmeB* mutant compared to wild type *C. jejuni* NCTC 11168. Bars represent the average of four biological replicates, and error bars represent standard deviation. Statistical significance as determined by an unpaired *t*-test is indicated by asterisks as follows: *p* < 0.05 (*), *p* < 0.01 (**).

Interestingly, we observed approximately two-fold upregulation of the entire *cmeABC* multidrug efflux pump at 30 and 120 min p.i. (Table 2), which has previously been shown to mediate *C. jejuni* resistance to antibiotics, ethidium bromide, and bile [58,59]. While multidrug efflux pumps serve as receptors for some phages [60], no previous link between multidrug efflux and phage infectivity has been established for *Campylobacter*. To test the hypothesis that the CmeABC system reduces phage infectivity in *C. jejuni* NCTC 11168, we assessed phage EOP on insertional mutants of *cmeA* and *cmeB*. Interestingly, we saw that the phage was able to infect *cmeA* (EOP = 2.00, *p* = 0.02) and *cmeB*-mutant cells (EOP = 2.26, *p* = 0.008) at higher levels than wild type cells (EOP = 1.00) (Figure 3). In addition to a reduction in EOP, plaques on *cmeA* and *cmeB* mutants were larger and clearer compared to those on the wild type strain.

#### 3.6.2. Oxidative Stress Defense May Affect *C. jejuni* Interactions with Phage NCTC 12673

We observed that the genes encoding the *chuABCD* heme transporter, along with the heme oxygenase *chuZ*, were among the most highly upregulated genes upon *C. jejuni* phage infection (+2–7-fold at most time points, Figure 2, Table 2). Additionally, several other iron uptake pathways were similarly upregulated (Table S5). These include genes involved in the transport of several siderophores, such as rhodotorulic acid (*cj1658, p19, cj1660–1662*), enterobactin (*cfrA*, *ceuB*), and lactoferrin/transferrin (*ctuA*) (Table S5) [61]. All of these genes are iron-repressed in *C. jejuni* NCTC 11168 [61]. In C*. jejuni*, iron homeostasis is closely linked to oxidative stress defense, as the main ferric uptake regulator in *C. jejuni*, Fur, also regulates many oxidative stress defense genes [41]. Interestingly, we also observed upregulation of the oxidative stress defense gene *katA* (+3-fold at 30 and 120 min p.i.). *katA* codes for the enzyme catalase, which catalyzes the dismutation of hydrogen peroxide to water and oxygen in *C. jejuni* and in most aerobic organisms [62]. *C. jejuni katA* mutants are known to be more sensitive to oxidative stresses [63]. It is noteworthy that *katA*, along with the *chuABCD* heme transporter, were both upregulated during infection, as KatA activity depends on heme [64].

**Figure 4:**
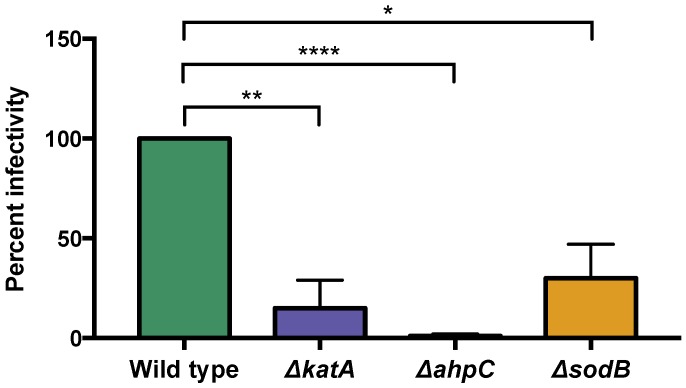
NCTC 12673 phage infection efficiency is reduced on a *katA* mutant, a *sodB* mutant, and an *ahpC* mutant compared to wild type *C. jejuni* NCTC 11168. Bars represent the average of three biological replicates, and error bars represent standard deviation. Statistical significance as determined by an unpaired *t*-test is indicated by asterisks as follows: *p* < 0.05 (*), *p* < 0.01 (**), *p* < 0.0001 (****).

To determine whether oxidative stress impacted NCTC 12673 infectivity, we determined the EOP of NCTC 12673 phage on a *katA* mutant. Interestingly, we found that the NCTC 12673 phage had an EOP of 0.15 (*p* = 0.009) on a *katA* mutant (Figure 4). To explore the effect of other types of oxidative stress on phage infection, we also tested EOP on two more mutants defective in oxidative stress defense, *ahpC*, which encodes alkyl-hydroxyperoxidase, and *sodB*, which encodes superoxide dismutase. We observed that both mutants showed lower EOP, with the *ahpC* mutation leading to an EOP of 0.01 (*p* < 0.0001) and *sodB* mutation resulting in an EOP of 0.30 (*p* = 0.02). Together, these results suggest that NCTC 12673 infectivity may be altered by multiple types of oxidative stresses (Figure 4).

#### 3.6.3. *cj0423–cj0425* Is the Most Highly Upregulated Host Operon upon Phage Infection

Interestingly, the host operon with the highest differential expression in response to phage infection across all time points was an uncharacterized operon comprised of *cj0423, cj0424*, and *cj0425*. These genes were upregulated between three- and 16-fold across all time points p.i. (Table 2). Although their functions have not been elucidated to date, *cj0423, cj0424*, and *cj0425* are predicted to encode a putative integral membrane protein, a putative acidic periplasmic protein and a putative periplasmic protein, respectively [17,18].

It has been previously reported that highly expressed gene clusters upon phage infection can be indicative of resident prophage expression [14,15]. Additionally, BLAST analysis showed that *cj0423–cj0425* is not universally conserved among *C. jejuni* strains. These features could indicate that *cj0423–cj0425* are prophage or cryptic prophage elements, but this is unlikely as previous work has suggested that the *C. jejuni* NCTC 11168 genome does not contain these features [17,18].

Instead, BLASTp analysis showed that the amino acid sequence of Cj0423 displays 40% identity (97% coverage, E value = 3 × 10^−14^) to the T4 superinfection immunity (Imm) protein (P08986) (Figure S3A), and both constitute members of the PFAM14373 Imm_superinfect family. Similar analysis of Cj0424 showed 36% identity (87% coverage, E value = 6 × 10^−8^) to the YwqK antitoxin component of the putative YwqJK toxin-antitoxin system in Bacillus subtilis strain 168 (Figure S3B) [65]. In contrast, BLASTp analysis did not reveal information about possible functions of *cj0425*.

## 4. Discussion

We used whole-transcriptome sequencing to better understand the gene expression program of phage NCTC 12673, a lytic T4-like myovirus with a 135-kB dsDNA genome most similar to T4-like cyanophages, and to characterize the response of *C. jejuni* NCTC 11168 to infection. Previous *C. jejuni* phage transcriptome experiments examined infection of the highly phage-susceptible strain *C. jejuni* PT14 (NCTC 12662) during carrier state association with the host [11]. We report the study of NCTC 12673 infection of *C. jejuni* NCTC 11168 as a host, which is notably not the propagating strain for this phage. The propagating strain, *C. jejuni* NCTC 12661, propagates NCTC 12673 to higher titres compared to NCTC 11168, for as-yet unknown reasons. However, NCTC 11168, being the first genome-sequenced strain of the species, has been much more extensively characterized. Thus, we chose to study the transcriptional profile of the NCTC 12673 infection of 11168 cells.

We performed our RNA-seq analysis on cells infected at a relatively low MOI (MOI = 1) compared to other phage-host RNA-seq studies, which have reported MOIs in the range of 10–25 [15]. However, the study of low-MOI infections, which are likely to be more representative of phage infections in nature, has lagged behind [66]. A high MOI allows for insights into phage transcriptional regulation to be gained, since nearly all cells in a culture are infected at once. In contrast, low MOI studies can lead to interesting insights into population-level host adaptation to phages, as phage-induced perturbations of both infected and uninfected neighbouring cells in a culture can be analyzed simultaneously. For instance, a recent study reported RNA-seq analysis of a *Staphylococcus aureus* phage infection at an MOI of 10^−5^, which uncovered phage-induced gene expression changes that led to increased biofilm production [67]. Phage-infected cells have been shown to delay lysis in response to the presence of infected sister cells nearby [45,46], and quorum sensing molecules have been shown to alter factors affecting phage susceptibility [14,48,68]. It is expected that other examples of phage-induced warning systems will be uncovered as more phage-host interactions are examined in detail [66]. It is thus becoming clear that studies examining phage infections at both high- and low-MOIs are independently useful for obtaining a comprehensive picture of phage-host dynamics.

Upon the infection of *C. jejuni* NCTC 11168 with NCTC 12673 phage, we did not observe the rapid reduction in relative host transcript abundance shortly after infection that has been observed for other phages [13,47]. However, since our infections were done at low MOI, our data do not preclude the possibility that NCTC 12673 degrades host transcripts on an individual cell level. Of note, NCTC 12673 does not appear to encode a homologue of the T4 phage *alc* gene, which is responsible for the near-immediate cessation of host transcription observed upon infection [47]. Therefore, if phage NCTC 12673 does inhibit host transcription, it likely does so by another mechanism. This study also allowed us to gain insight into the host genes that might be involved in phage DNA, RNA, and protein synthesis during *C. jejuni* infection. In line with expectations for this phage, which does not encode its own RNA polymerase, we observed that host RNA polymerase genes were upregulated upon infection. Unexpectedly, genes like host DNA polymerase III and ribonucleotide reductases, versions of which are also encoded by the phage, were also upregulated. It remains to be determined to what extent the phage- vs. host-derived versions of these genes function in phage replication, but it is interesting to speculate that both versions might play roles in phage replication.

By analyzing phage transcripts, we were able to validate previously predicted transcriptional start sites, termination sites, and promoters, as well as identify two putative promoter motifs. We observed the expression of most NCTC 12673 coding sequences predicted by Kropinski et al., and our analysis revealed that many of the proteins detected in the NCTC 12673 structural proteome tended to be more highly expressed relative to other phage genes [25]. The majority of predicted NCTC 12673 ORFs lack assigned functions, and our validation of previous genomic and proteomic studies of this phage should assist in guiding further studies toward elucidating these functions.

As transcriptional analysis of phage-host interactions has become more common, many phages have now been shown to induce differential gene expression in their hosts. For instance, cyanophages have been shown to alter the expression of host metabolic genes and stress responses [9] and the *Pseudomonas* phage PAK_P4 has been suggested to alter the expression of host iron-scavenging genes in *P. aeruginosa* [15]. We observed the differential expression of many genes involved in other metabolic functions in the host cell, such as genes involved in amino acid metabolism, oxidative stress defense, iron homeostasis, and multi-drug efflux pump production, as well as several uncharacterized genes.

We observed the differential expression of several amino acid pathways upon phage infection, including the upregulation of genes involved in asparagine, leucine, isoleucine, and valine metabolism, and the downregulation of components involved in aspartate, glutamate, serine, and proline metabolism. *C. jejuni* is known to tightly regulate its amino acid metabolism, and has been shown to metabolize amino acids sequentially in order of preference, beginning with serine, followed by aspartate, asparagine, and glutamate [69]. It is possible that NCTC 12673 phage infection may have different amino acid requirements compared to the uninfected cell, perhaps leading it to induce changes in gene expression to procure the amino acids it requires. It is particularly interesting that three of the four amino acids most preferentially utilized by *C. jejuni* are downregulated upon phage NCTC 12673 infection.

Phage nutritional requirements have not yet been well studied, though recent metabolomics studies have documented phage-specific changes in amino acid metabolism for several *Pseudomonas* phages and a *Roseobacter* phage [48,49]. Additionally, an earlier study showed that the lambda phage requires the branched chain amino acids leucine, isoleucine, valine, and threonine [70]. Amino acid availability was shown to affect T4 lytic infection of stationary phase *E. coli,* although specific amino acids important for phage propagation were not identified [71]. While changes in amino acid metabolism could represent a phage-directed response, it has also been suggested that phage-infected cells may alter amino acid metabolism as a way of limiting phage infection [14,48]. Further work should seek to understand whether NCTC 12673 phage requires specific amino acids for infection, and whether the phage and/or host manipulates these stores to their own advantage.

Interestingly, several host genes involved in inorganic ion transport and metabolism were upregulated upon NCTC 12673 phage infection. In particular, the heme-related genes *chuABCD* and *chuZ,* as well as the heme-dependent oxidative stress defense enzyme *katA,* were among the most highly upregulated. Upregulation of iron acquisition genes has been observed in other phage-host systems, such as PAK_P4 infection of *P. aeruginosa*, but this phenomenon is not well understood [15]. Of note, phage PAK_P3, a different but related *P. aeruginosa* phage, does not induce iron uptake gene expression in the same host strain, suggesting that PAK_P4 iron uptake may be phage-directed as opposed to host-directed [15].

The presence of iron leads to increased oxidative stress sensitivity in *C. jejuni,* and the regulation of responses to oxidative stress and iron levels are highly intertwined in this organism [41]. We therefore hypothesized that phage infection-induced upregulation of iron uptake systems might lead to oxidative stress sensitivity in the cells, and that this might alter NCTC 12673 phage infection efficiency. To test the effect of oxidative stress sensitivity on phage infection efficiency, we examined whether the phage could efficiently infect mutants defective in three oxidative stress defense genes: *katA*, *ahpC*, and *sodB*. *ahpC* encodes alkyl-hydroxyperoxidase, and although catalase is the primary detoxifier of H_2_O_2_ in *C. jejuni*, AhpC is also capable of converting H_2_O_2_ and organic hydroperoxides (ROOH) to their corresponding alcohols. *sodB* encodes a superoxide dismutase, which converts superoxide to hydrogen peroxide. Interestingly, we observed a reduction in phage infectivity on all three mutants, suggesting that oxidative stress negatively affects NCTC 12673 phage infection.

Neither *ahpC* or *sodB* were upregulated upon phage infection, which may be related to the fact that *katA* is co-regulated by the *C. jejuni* ferric uptake regulator (Fur), while *ahpC* and *sodB* are not [41]. Given the observed importance of oxidative stress defense genes for NCTC 12673 phage infection and the fact that many classical members of the Fur regulon are dysregulated during infection, our data suggests that iron homeostasis may be the initial perturbation upon phage infection, and that oxidative stress may follow later. It is unknown why iron uptake pathways would be upregulated upon phage infection, particularly as our infections were done in iron-replete media.

With the exception of the *mcrC* component of an McrBC 5-methylcytosine restriction system at one time point p.i., we did not observe host upregulation of restriction enzymes or CRISPR-Cas genes in response to NCTC 12673 phage infection. *C. jejuni* encodes a Type II-C CRISPR-Cas system, and Dugar *et al.* found that CRISPR regions were among the most highly transcribed loci in *C. jejuni* NCTC 11168 [72]. Although carrier state-associated *C. jejuni* phages were shown to induce host-directed spacer acquisition, *C. jejuni* CRISPR-Cas has not yet been shown to function in phage defense [53]. Others have identified an inverse correlation between *C. jejuni* strains that encode plasmids, prophages, or other phage defense mechanisms and those that encode CRISPR-Cas systems, suggesting a possible role of CRISPR-Cas in phage defense [72,73]. It has also been suggested that *Campylobacter* CRISPR-Cas evolved a function unrelated to phage defense, perhaps to regulate its own genes, as has been shown for other organisms [73,74,75]. Very recently, *Campylobacter* CRISPR-Cas systems were shown to specifically target endogenous mRNA and ssRNA, supporting this possibility [74,76].

We also observed the upregulation of genes involved in MeOPN addition to CPS upon phage infection. Notably, we and others have previously shown that changes in MeOPN on *C. jejuni* CPS affect the susceptibility to several other *Campylobacter* phages, and that phage pressure can lead to changes in expression of these structures through slipped strand mispairing in phase-variable genes [31,77,78,79]. Our observation that NCTC 12673 phage pressure induces changes in the transcription of CPS biosynthesis genes suggests another possible mechanism by which *C. jejuni* might alter its CPS in response to phage infection, but further work is required to determine whether changes in CPS gene transcription might affect susceptibility to phages.

Although canonical phage defense systems were not upregulated in response to NCTC 12673 infection, we observed upregulation of the multidrug efflux pump genes *cmeABC* and found that *cmeA* and *cmeB* efflux pump mutants were more efficiently infected by NCTC 12673. This suggests a possible role of this efflux pump in phage defense. It could be hypothesized that an efflux pump might function to pump out the metabolites that a phage requires for efficient infection (i.e., similar to a “scorched earth” strategy, whereby an infected cell depletes phages of necessary resources to reduce infection efficiency) [14,80]. Alternatively, an efflux pump could function in phage resistance by pumping out factors that might alert sister cells of an imminent phage threat. Others have observed the upregulation of *P. aeruginosa* multidrug efflux pump genes in response to phage infection [14], but to our knowledge, efflux pumps have not been shown to function in phage defense. Some phages have been shown to require efflux pumps to bind host cells [81], and increased resistance to antibiotics has also been described in response to phage infection [66,82,83]. It is intriguing to speculate that phage-induced expression of efflux pumps might increase antibiotic resistance under some conditions. Further work into understanding the influence of efflux pumps like *cmeABC* on phage infection and antibiotic susceptibility is thus required, as knowledge in this area is particularly relevant for the safe administration of therapeutic phages.

Interestingly, we observed significant upregulation of the uncharacterized operon *cj0423–cj0425* in response to phage infection*,* which comprises one gene (*cj0423)* encoding a protein homologous to the T4 superinfection immunity protein Imm*,* and another (*cj0424*) encoding a protein homologous to an antitoxin component of the RNAse-mediated toxin-antitoxin system YwqJK [65]. Imm is a component of one of two T4 superinfection exclusion (Sie) systems, and has been shown to block T-even phage DNA injection into *Escherichia coli* cells [47,84,85]. Furthermore, it should be noted that toxin-antitoxin systems have been shown to function in anti-phage abortive infection systems [86]. The last gene of this operon, *cj0425*, displays no homology to phage defense genes. However, others have speculated that *cj0425* may contribute to oxidative stress tolerance based on its identification as one of two *C. jejuni* NCTC 11168 genes with a CXXC redox motif not present in the more oxygen-susceptible strain *C. jejuni* RM1221 [87]. Others have shown that *cj0425* is downregulated upon low-oxygen in vitro growth and chick colonization of *C. jejuni* [87,88,89], supporting a possible role in oxygen tolerance. It is intriguing that this last gene appears to be involved in oxidative stress, considering the importance of oxidative stress for NCTC 12673 infectivity that we have described here.

It is possible that this operon represents a phage exclusion system that may help explain the reduced phage EOP on *C. jejuni* NCTC 11168 compared to other strains. In support of this hypothesis, this operon is not encoded by the propagating strain for this phage, *C. jejuni* NCTC 12661, which is more efficiently infected by NCTC 12673. Further work is required to determine whether *cj0423–cj0425* expression prevents or reduces phage infection in *C. jejuni.* It is also of interest to identify whether this operon is induced by phage-infected cells and/or by uninfected cells in the vicinity of phage infection. If this operon does function in phage defense, it is perhaps more likely that uninfected cells rather than infected cells would express this operon. However, not all phage defense systems serve to completely abolish phage infection, and certain defense mechanisms are proposed to reduce infection vigour without necessarily abolishing it [14,90]. Notably, our results do not rule out either possibility.

Due to the lack of a stable reference transcript maintained by the host at a consistent copy number under uninfected and infected conditions, we validated our RNA-seq results with mutagenesis and EOP assays rather than by the historically used RT-qPCR. Importantly, apart from the genes verified here for their importance in phage infection (*sodB*, *ahpC*, *katA*, *cmeA*, and *cmeB*), the other gene expression changes highlighted in this study should be verified experimentally prior to drawing conclusions about their involvement in phage infection.

The changes in host gene expression upon infection that we have observed can suggest either a host response to the phage, phage modulation of host genes, or a combination of both. To distinguish between whether changes in gene expression are phage- or host-induced, analysis of the same phage on multiple hosts, and/or analysis of multiple phages on the same host, should be conducted [12,14,15]. To determine whether observed changes in gene expression occur in infected and/or uninfected hosts, RNA-seq of synchronously-infected cells could be pursued to effectively eliminate the detection of RNA from uninfected cells. Overall, whether the observed phage infection-induced transcriptional responses are driven by phages, by infected hosts, or by uninfected hosts, RNA-seq analysis of this complex interplay is a useful first step toward generating important new data about phage-host interactions.

## 5. Conclusions

This work represents the first reported whole-transcriptome sequencing analysis of *C. jejuni* during lytic phage infection. Little is known about the determinants of phage susceptibility among different *C. jejuni* strains, and yet, understanding these determinants is essential to the success of phage-mediated biocontrol of *C. jejuni.* Furthermore, understanding the factors governing the phage-host dynamics of any bacterial species can lead to important insights into its evolution. Finally, this analysis represents one of the first RNA-seq-based analyses of a low MOI phage infection, and has highlighted several genes that may play roles in population-wide *C. jejuni* responses to phage infection.

## Figures and Tables

**Table 1:** List of bacterial strains and phages used in this study.

Name	Source	Reference
*C. jejuni* NCTC 11168 MP21	Chicken	[17,18,31]
Phage NCTC 12673	Chicken	[24,25]
*C. jejuni* NCTC 11168 ∆*katA*	*C. jejuni* NCTC 11168	[41]
*C. jejuni* NCTC 11168 ∆*ahpC*	*C. jejuni* NCTC 11168	[41]
*C. jejuni* NCTC 11168 ∆*sodB*	*C. jejuni* NCTC 11168	[41]
*C. jejuni* NCTC 11168 ∆*cmeA*	*C. jejuni* NCTC 11168	Patry et al. submitted [42]
*C. jejuni* NCTC 11168 ∆*cmeB*	*C. jejuni* NCTC 11168	[43]

**Table 2:** Fold changes in gene expression for selected *C. jejuni* NCTC 11168 genes at 30, 60, and 120 min post NCTC 12673 phage addition compared to mock-infected controls at each time point.

		Fold Change (*P*-Value) ^a^		
Gene	Annotation	30 min (*p*)	60 min (*p*)	120 min (*p*)
Multi-drug efflux pump CmeABC				
*cmeA*	Periplasmic fusion protein CmeA (multidrug efflux system CmeABC)	2.6 (<0.01)	NS	1.99 (<0.01)
*cmeB*	Inner membrane efflux transporter CmeB (multidrug efflux system CmeABC)	2.09 (<0.01)	NS	2.14 (<0.01)
*cmeC*	Outer membrane channel protein CmeC (multidrug efflux system CmeABC)	2.04 (<0.01)	NS	2.02 (<0.01)
Oxidative stress and iron metabolism				
*katA*	Catalase	3.1 (<0.01)	NS	3.42 (<0.01)
*chuA*	Haemin uptake system outer membrane receptor	7.73 (<0.01)	4.9 (<0.01)	6.84 (<0.01)
*chuB*	Putative haemin uptake system permease protein	3.02 (<0.01)	NS	1.8 (0.02)
*chuC*	Putative haemin uptake system ATP-binding protein	NS	NS	2.49 (0.05)
*chuD*	Putative haemin uptake system periplasmic haemin-binding protein	3.9 (<0.01)	2.73 (0.02)	3.31 (<0.01)
*chuZ^b^*	Iron-responsive cellular heme oxygenase	3.34 (<0.01)	3.25 (<0.01)	2.47 (<0.01)
Uncharacterized operon *cj0423–cj0425*				
*cj0423*	Putative integral membrane protein	5.73 (<0.01)	3.36 (<0.01)	16.14 (<0.01)
*cj0424*	Putative acidic periplasmic protein	9.95 (<0.01)	10.51 (<0.01)	12.75 (<0.01)
*cj0425*	Putative periplasmic protein	9.01 (<0.01)	9.1 (<0.01)	10.39 (<0.01)

^a^ Genes were considered differentially expressed when the false discovery rate-corrected *p*-value < 0.05. NS = not statistically significant, cells were left blank. ^b^ Annotation updated according to [57].

## Supplementary Materials

The following are available online at http://www.mdpi.com/1999-4915/10/6/332/s1, Figure S1. Principal component analysis plot for infected vs. uninfected controls at each time point post NCTC 12673 phage infection of *C. jejuni* NCTC 11168 cells. Figure S2. Nucleotide sequence of the novel 158-bp non-coding RNA (ncRNA) identified between *gp012* and *gp013* (nucleotides 7110-7267) on the phage genome. Figure S3. BLASTp alignment between *C. jejuni* NCTC 11168 protein Cj0423 and T4 phage protein Imm (A) and between *C. jejuni* NCTC 11168 protein Cj0424 and *Bacillus subtilis* strain 168 antitoxin protein YwqK (B). Table S1. RNA-seq read statistics. Table S2. Relative transcription of NCTC 12673 phage genes during *C. jejuni* NCTC 11168 infection. Table S3. Number and percentage of *C. jejuni* NCTC 11168 genes differentially expressed (DE) at each time point post NCTC 12673 phage infection. Table S4. KEGG pathways for *C. jejuni* NCTC 11168 genes statistically significantly downregulated at 30, 60, and 120 min post NCTC 12673 phage infection. Table S5. Differentially expressed *C. jejuni* NCTC 11168 genes upon NCTC 12673 phage infection according to COG categories.

## References

[1] O’Neill J. (2014). Antimicrobial Resistance: Tackling a Crisis for the Health and Wealth of Nations.

[2] Huys I., Pirnay J.P., Lavigne R., Jennes S., de Vos D., Casteels M., Verbeken G. (2013). Paving a regulatory pathway for phage therapy. Europe should muster the resources to financially, technically and legally support the introduction of phage therapy. EMBO Rep..

[3] Roach D.R., Debarbieux L. (2017). Phage therapy: Awakening a sleeping giant. Emerg. Top. Life Sci..

[4] Umaraw P., Prajapati A., Verma A.K., Pathak V., Singh V.P. (2017). Control of *Campylobacter* in poultry industry from farm to poultry processing unit: A review. Crit. Rev. Food Sci. Nutr..

[5] Blasdel B., Ceyssens P.-J., Lavigne R. (2018). Preparing cDNA Libraries from Lytic Phage-Infected Cells for Whole Transcriptome Analysis by RNA-Seq. Methods Molecular Biology.

[6] Leskinen K., Blasdel B.G., Lavigne R., Skurnik M. (2016). RNA-sequencing reveals the progression of Phage-Host interactions between φR1-37 and *Yersinia enterocolitica*. Viruses.

[7] Ceyssens P.-J., Minakhin L., Van den Bossche A., Yakunina M., Klimuk E., Blasdel B., de Smet J., Noben J.-P., Bläsi U., Severinov K. (2014). Development of giant bacteriophage ϕKZ is independent of the host transcription apparatus. J. Virol..

[8] Morimoto D., Kimura S., Sako Y., Yoshida T. (2018). Transcriptome Analysis of a Bloom-Forming Cyanobacterium *Microcystis aeruginosa* during Ma-LMM01 Phage Infection. Front. Microbiol..

[9] Doron S., Fedida A., Hernández-Prieto M.A., Sabehi G., Karunker I., Stazic D., Feingersch R., Steglich C., Futschik M., Lindell D. (2016). Transcriptome dynamics of a broad host-range cyanophage and its hosts. ISME J..

[10] Mojardín L., Salas M. (2016). Global transcriptional analysis of virus-host interactions between phage φ29 and *Bacillus subtilis*. J. Virol..

[11] Brathwaite K.J., Siringan P., Connerton P.L., Connerton I.F. (2015). Host adaption to the bacteriophage carrier state of *Campylobacter jejuni*. Res. Microbiol..

[12] Howard-Varona C., Roux S., Dore H., Solonenko N.E., Holmfeldt K., Markillie L.M., Orr G., Sullivan M.B. (2017). Regulation of infection efficiency in a globally abundant marine Bacteriodetes virus. ISME J..

[13] Chevallereau A., Blasdel B.G., De Smet J., Monot M., Zimmermann M., Kogadeeva M., Sauer U., Jorth P., Whiteley M., Debarbieux L. (2016). Next-Generation “-omics” Approaches Reveal a Massive Alteration of Host RNA Metabolism during Bacteriophage Infection of Pseudomonas aeruginosa. PLoS Genet..

[14] Blasdel B.G., Ceyssens P.-J., Chevallereau A., Debarbieux L., Lavigne R. (2018). Comparative transcriptomics reveals a conserved Bacterial Adaptive Phage Response (BAPR) to viral predation. bioRxiv.

[15] Blasdel B.G., Chevallereau A., Monot M., Lavigne R., Debarbieux L. (2017). Comparative transcriptomics analyses reveal the conservation of an ancestral infectious strategy in two bacteriophage genera. ISME J..

[16] Lin X., Ding H., Zeng Q. (2016). Transcriptomic response during phage infection of a marine cyanobacterium under phosphorus-limited conditions. Environ. Microbiol..

[17] Parkhill J., Wren B.W., Mungall K., Ketley J.M., Churcher C., Basham D., Chillingworth T., Davies R.M., Feltwell T., Holroyd S. (2000). The genome sequence of the food-borne pathogen *Campylobacter jejuni* reveals hypervariable sequences. Nature.

[18] Gundogdu O., Bentley S.D., Holden M.T., Parkhill J., Dorrell N., Wren B.W. (2007). Re-annotation and re-analysis of the *Campylobacter jejuni* NCTC11168 genome sequence. BMC Genom..

[19] Kaakoush N.O., Mitchell H.M., Man S.M. (2015). Campylobacter. Molecular Medical Microbiology.

[20] Wassenaar T.M. (2011). Following an imaginary *Campylobacter* population from farm to fork and beyond: A bacterial perspective. Lett. Appl. Microbiol..

[21] Zampara A., Sørensen M.C.H., Elsser-Gravesen A., Brøndsted L. (2017). Significance of phage-host interactions for biocontrol of *Campylobacter jejuni* in food. Food Control.

[22] Fischer S., Kittler S., Klein G., Glünder G. (2013). Impact of a Single Phage and a Phage Cocktail Application in Broilers on Reduction of *Campylobacter jejuni* and Development of Resistance. PLoS ONE.

[23] Kittler S., Fischer S., Abdulmawjood A., Glünder G., Kleina G. (2013). Effect of bacteriophage application on *Campylobacter jejuni* loads in commercial broiler flocks. Appl. Environ. Microbiol..

[24] Grajewski B.A., Kusek J.W., Gelfand H.M. (1985). Development of bacteriophage typing system for *Campylobacter jejuni* and *Campylobacter coli*. J. Clin. Microbiol..

[25] Kropinski A.M., Arutyunov D., Foss M., Cunningham A., Ding W., Singh A., Pavlov A.R., Henry M., Evoy S., Kelly J. (2011). Genome and proteome of *Campylobacter jejuni* bacteriophage NCTC 12673. Appl. Environ. Microbiol..

[26] Javed M.A., Ackermann H.W., Azeredo J., Carvalho C.M., Connerton I., Evoy S., Hammerl J.A., Hertwig S., Lavigne R., Singh A. (2014). A suggested classification for two groups of *Campylobacter* myoviruses. Arch. Virol..

[27] Sørensen M.C.H., Gencay Y.E., Birk T., Baldvinsson S.B., Jäckel C., Hammerl J.A., Vegge C.S., Neve H., Brøndsted L. (2015). Primary isolation strain determines both phage type and receptors recognised by *Campylobacter jejuni* bacteriophages. PLoS ONE.

[28] Coward C., Grant A.J., Swift C., Philp J., Towler R., Heydarian M., Frost J.A., Maskell D.J. (2006). Phase-variable surface structures are required for infection of *Campylobacter jejuni* by bacteriophages. Appl. Environ. Microbiol..

[29] Javed M.A., van Alphen L.B., Sacher J., Ding W., Kelly J., Nargang C., Smith D.F., Cummings R.D., Szymanski C.M. (2015). A receptor-binding protein of *Campylobacter jejuni* bacteriophage NCTC 12673 recognizes flagellin glycosylated with acetamidino-modified pseudaminic acid. Mol. Microbiol..

[30] Javed M.A., Sacher J.C., van Alphen L.B., Patry R.T., Szymanski C.M. (2015). A flagellar glycan-specific protein encoded by *Campylobacter* phages inhibits host cell growth. Viruses.

[31] Sørensen M.C.H., van Alphen L.B., Harboe A., Li J., Christensen B.B., Szymanski C.M., Brøndsted L. (2011). Bacteriophage F336 recognizes the capsular phosphoramidate modification of *Campylobacter jejuni* NCTC11168. J. Bacteriol..

[32] Frost J.A., Kramer J.M., Gillanders S.A. (1999). Phage typing of *Campylobacter jejuni* and *Campylobacter coli* and its use as an adjunct to serotyping. Epidemiol. Infect..

[33] Sørensen M.C.H., Gencay Y.E., Brøndsted L. (2017). Methods for initial characterization of *Campylobacter jejuni* bacteriophages. Methods Mol. Biol..

[34] Palyada K., Threadgill D., Stintzi A. (2004). Iron acquisition and regulation in *Campylobacter jejuni*. J. Bacteriol..

[35] Love M.I., Huber W., Anders S. (2014). Moderated estimation of fold change and dispersion for RNA-seq data with DESeq2. Genome Biol..

[36] Dobin A., Davis C.A., Schlesinger F., Drenkow J., Zaleski C., Jha S., Batut P., Chaisson M., Gingeras T.R. (2013). STAR: Ultrafast universal RNA-seq aligner. Bioinformatics.

[37] Kanehisa M., Furumichi M., Tanabe M., Sato Y., Morishima K. (2017). KEGG: New perspectives on genomes, pathways, diseases and drugs. Nucleic Acids Res..

[38] Luo W., Friedman M.S., Shedden K., Hankenson K.D., Woolf P.J. (2009). GAGE: Generally applicable gene set enrichment for pathway analysis. BMC Bioinform..

[39] Bailey T.L., Elkan C. (1994). Fitting a mixture model by expectation maximization to discover motifs in biopolymers. Proc. Int. Conf. Intell. Syst. Mol. Biol..

[40] Grant C.E., Bailey T.L., Noble W.S. (2011). FIMO: scanning for occurrences of a given motif. Bioinformatics.

[41] Palyada K., Sun Y.Q., Flint A., Butcher J., Naikare H., Stintzi A. (2009). Characterization of the oxidative stress stimulon and PerR regulon of *Campylobacter jejuni*. BMC Genom..

[42] Patry R.T., Stahl M., Perez-Munoz M.E., Nothaft H., Wenzel C.Q., Sacher J.C., Coros C., Walter J., Vallance B.A., Szymanski C.M. (2018). Bacterial warfare: growth inhibition of ganglioside-mimicking gut bacteria by AB5 toxins. Nat. Microbiol..

[43] Akiba M., Lin J., Barton Y.-W., Zhang Q. (2006). Interaction of CmeABC and CmeDEF in conferring antimicrobial resistance and maintaining cell viability in *Campylobacter jejuni*. J. Antimicrob. Chemother..

[44] Loc Carrillo C., Atterbury R.J., El-Shibiny A., Connerton P.L., Dillon E., Scott A., Connerton I.F. (2005). Bacteriophage therapy to reduce *Campylobacter jejuni* colonization of broiler chickens. Appl. Environ. Microbiol..

[45] Erez Z., Steinberger-Levy I., Shamir M., Doron S., Stokar-Avihail A., Peleg Y., Melamed S., Leavitt A., Savidor A., Albeck S. (2017). Communication between viruses guides lysis-lysogeny decisions. Nature.

[46] Abedon S.T. (2017). Commentary: Communication between Viruses Guides Lysis-Lysogeny Decisions. Front. Microbiol..

[47] Miller E.S., Kutter E., Mosig G., Arisaka F., Kunisawa T., Rüger W. (2003). Bacteriophage T4 genome. Microbiol. Mol. Biol. Rev..

[48] De Smet J., Zimmermann M., Kogadeeva M., Ceyssens P.J., Vermaelen W., Blasdel B., Bin Jang H., Sauer U., Lavigne R. (2016). High coverage metabolomics analysis reveals phage-specific alterations to *Pseudomonas aeruginosa* physiology during infection. ISME J..

[49] Ankrah N.Y.D., May A.L., Middleton J.L., Jones D.R., Hadden M.K., Gooding J.R., LeCleir G.R., Wilhelm S.W., Campagna S.R., Buchan A. (2014). Phage infection of an environmentally relevant marine bacterium alters host metabolism and lysate composition. ISME J..

[50] Guccione E., del Rocio Leon-Kempis M., Pearson B.M., Hitchin E., Mulholland F., van Diemen P.M., Stevens M.P., Kelly D.J. (2008). Amino acid-dependent growth of *Campylobacter jejuni* : key roles for aspartase (AspA) under microaerobic and oxygen-limited conditions and identification of AspB (Cj0762), essential for growth on glutamate. Mol. Microbiol..

[51] Sacher J.C. (2017). Campylobacter Phage Propagating Strain NCTC 12661 Propagates Phage NCTC 12673 More Efficiently Than Does Strain NCTC 11168.

[52] Anjum A., Brathwaite K.J., Aidley J., Connerton P.L., Cummings N.J., Parkhill J., Connerton I., Bayliss C.D. (2016). Phase variation of a Type IIG restriction-modification enzyme alters site-specific methylation patterns and gene expression in *Campylobacter jejuni* strain NCTC11168. Nucleic Acids Res..

[53] Hooton S.P.T., Connerton I.F. (2015). *Campylobacter jejuni* acquire new host-derived CRISPR spacers when in association with bacteriophages harboring a CRISPR-like Cas4 protein. Front. Microbiol..

[54] Gardner S.P., Olson J.W. (2012). Barriers to Horizontal Gene Transfer in *Campylobacter jejuni*. Adv. Appl. Microbiol..

[55] Louwen R., van Baarlen P. (2013). Are bacteriophage defence and virulence two sides of the same coin in *Campylobacter jejuni*?. Biochem. Soc. Trans..

[56] McNally D.J., Lamoureux M.P., Karlyshev A.V., Fiori L.M., Li J., Thacker G., Coleman R.A., Khieu N.H., Wren B.W., Brisson J.R. (2007). Commonality and biosynthesis of the O-methyl phosphoramidate capsule modification in *Campylobacter jejuni*. J. Biol. Chem..

[57] Ridley K.A., Rock J.D., Li Y., Ketley J.M. (2006). Heme utilization in *Campylobacter jejuni*. J. Bacteriol..

[58] Lin J., Michel L.O., Zhang Q. (2002). CmeABC functions as a multidrug efflux system in *Campylobacter jejuni*. Antimicrob. Agents Chemother..

[59] Pumbwe L., Piddock L.J.V. (2002). Identification and molecular characterisation of CmeB, a *Campylobacter jejuni* multidrug efflux pump. FEMS Microbiol. Lett..

[60] De Smet J., Hendrix H., Blasdel B.G., Danis-Wlodarczyk K., Lavigne R. (2017). *Pseudomonas* predators: Understanding and exploiting phage-host interactions. Nat. Rev. Microbiol..

[61] Butcher J., Stintzi A. (2013). The Transcriptional Landscape of *Campylobacter jejuni* under Iron Replete and Iron Limited Growth Conditions. PLoS ONE.

[62] Grant K.A., Park S.F. (1995). Molecular characterization of *katA* from *Campylobacter jejuni* and generation of a catalase-deficient mutant of *Campylobacter coli* by interspecific allelic exchange. Microbiology.

[63] Day W.A., Sajecki J.L., Pitts T.M., Joens L.A., Joens L.A. (2000). Role of catalase in *Campylobacter jejuni* intracellular survival. Infect. Immun..

[64] Flint A., Sun Y.Q., Stintzi A. (2012). Cj1386 is an ankyrin-containing protein involved in HEME trafficking to catalase in *Campylobacter jejuni*. J. Bacteriol..

[65] Holberger L.E., Garza-Sánchez F., Lamoureux J., Low D.A., Hayes C.S. (2012). A novel family of toxin/antitoxin proteins in *Bacillus* species. FEBS Lett..

[66] Fernández L., Rodríguez A., García P. (2018). Phage or foe: an insight into the impact of viral predation on microbial communities. ISME J..

[67] Fernández L., González S., Campelo A.B., Martínez B., Rodríguez A., García P. (2017). Low-level predation by lytic phage phiIPLA-RODI promotes biofilm formation and triggers the stringent response in *Staphylococcus aureus*. Sci. Rep..

[68] Moreau P., Diggle S.P., Friman V.-P. (2017). Bacterial cell-to-cell signaling promotes the evolution of resistance to parasitic bacteriophages. Ecol. Evol..

[69] Stahl M., Butcher J., Stintzi A. (2012). Nutrient acquisition and metabolism by *Campylobacter jejuni*. Front. Cell. Infect. Microbiol..

[70] Gots J.S., Hunt G.R. (1953). Amino acid requirements for the maturation of bacteriophage in lysogenic *Escherichia coli*. J. Bacteriol..

[71] Bryan D., El-Shibiny A., Hobbs Z., Porter J., Kutter E.M. (2016). Bacteriophage T4 infection of stationary phase *E. coli*: Life after log from a phage perspective. Front. Microbiol..

[72] Dugar G., Herbig A., Förstner K.U., Heidrich N., Reinhardt R., Nieselt K., Sharma C.M. (2013). High-Resolution Transcriptome Maps Reveal Strain-Specific Regulatory Features of Multiple *Campylobacter jejuni* Isolates. PLoS Genet..

[73] Louwen R., Horst-Kreft D., de Boer A.G., Van Der Graaf L., De Knegt G., Hamersma M., Heikema A.P., Timms A.R., Jacobs B.C., Wagenaar J.A. (2013). A novel link between *Campylobacter jejuni* bacteriophage defence, virulence and Guillain-Barre syndrome. Eur. J. Clin. Microbiol. Infect. Dis..

[74] Dugar G., Leenay R.T., Eisenbart S.K., Bischler T., Aul B.U., Beisel C.L., Sharma C.M. (2018). CRISPR RNA-Dependent Binding and Cleavage of Endogenous RNAs by the *Campylobacter jejuni* Cas9. Mol. Cell.

[75] Li R., Fang L., Tan S., Yu M., Li X., He S., Wei Y., Li G., Jiang J., Wu M. (2016). Type I CRISPR-Cas targets endogenous genes and regulates virulence to evade mammalian host immunity. Cell Res..

[76] Strutt S.C., Torrez R.M., Kaya E., Negrete O.A., Doudna J.A. (2018). RNA-dependent RNA targeting by CRISPR-Cas9. eLife.

[77] Sørensen M.C.H., van Alphen L.B., Fodor C., Crowley S.M., Christensen B.B., Szymanski C.M., Brøndsted L. (2012). Phase Variable Expression of Capsular Polysaccharide Modifications Allows *Campylobacter jejuni* to Avoid Bacteriophage Infection in Chickens. Front. Cell. Infect. Microbiol..

[78] Aidley J., Holst Sørensen M.C., Bayliss C.D., Brøndsted L. (2017). Phage exposure causes dynamic shifts in the expression states of specific phase-variable genes of *Campylobacter jejuni*. Microbiology.

[79] Gencay Y.E., Sørensen M.C.H., Wenzel C.Q., Szymanski C.M., Brøndsted L. (2018). Phase Variable Expression of a Single Phage Receptor in *Campylobacter jejuni* NCTC12662 Influences Sensitivity Toward Several Diverse CPS-Dependent Phages. Front. Microbiol..

[80] Hyman P., Abedon S.T. (2010). Bacteriophage host range and bacterial resistance. Adv. Appl. Microbiol..

[81] Chan B.K., Sistrom M., Wertz J.E., Kortright K.E., Narayan D., Turner P.E. (2016). Phage selection restores antibiotic sensitivity in MDR *Pseudomonas aeruginosa*. Sci. Rep..

[82] Wang X., Kim Y., Ma Q., Hong S.H., Pokusaeva K., Sturino J.M., Wood T.K. (2010). Cryptic prophages help bacteria cope with adverse environments. Nat. Commun..

[83] Schuch R., Fischetti V.A. (2006). Detailed genomic analysis of the Wbeta and gamma phages infecting *Bacillus anthracis*: implications for evolution of environmental fitness and antibiotic resistance. J. Bacteriol..

[84] Lu M.J., Henning U. (1989). The immunity (IMM) gene of *Escherichia coli* bacteriophage T4. J. Virol..

[85] Labrie S.J., Samson J.E., Moineau S. (2010). Bacteriophage resistance mechanisms. Nat. Rev. Microbiol..

[86] Fineran P.C., Blower T.R., Foulds I.J., Humphreys D.P., Lilley K.S., Salmond G.P.C. (2009). The phage abortive infection system, ToxIN, functions as a protein-RNA toxin-antitoxin pair. Proc. Natl. Acad. Sci. USA.

[87] Kaakoush N.O., Miller W.G., De Reuse H., Mendz G.L. (2007). Oxygen requirement and tolerance of *Campylobacter jejuni*. Res. Microbiol..

[88] Woodall C.A., Jones M.A., Barrow P.A., Hinds J., Marsden G.L., Kelly D.J., Dorrell N., Wren B.W., Maskell D.J. (2005). *Campylobacter jejuni* gene expression in the chick cecum: Evidence for adaptation to a low-oxygen environment. Infect. Immun..

[89] Gaynor E.C., Cawthraw S., Manning G., MacKichan J.K., Falkow S., Newell D.G. (2004). The genome-sequenced variant of *Campylobacter jejuni* NCTC 11168 and the original clonal clinical isolate differ markedly in colonization, gene expression, and virulence-associated phenotypes. J. Bacteriol..

[90] Ofir G., Melamed S., Sberro H., Mukamel Z., Silverman S., Yaakov G., Doron S., Sorek R. (2018). DISARM is a widespread bacterial defence system with broad anti-phage activities. Nat. Microbiol..

