# Supplementary material for: Transcriptomic Analysis of the Campylobacter jejuni Response to T4-Like Phage NCTC 12673 Infection
Source: Viruses. 2018 Jun 16;10(6):332. doi: 10.3390/v10060332 (PMC6024767; doi:10.3390/v10060332)
Supplement: Supplementary file 1 [file viruses-10-00332-s001.pdf]

Supplementary materials

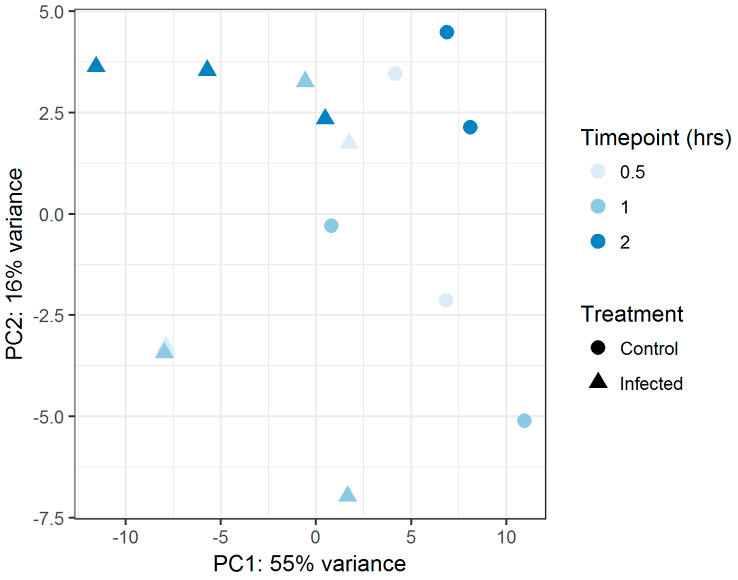

Figure S1. PCA plot for infected vs. uninfected controls at each time point post NCTC 12673 phage infection of *C. jejuni* NCTC 11168 cells. Plots were generated using the data from the 500 genes with the greatest variation in expression across samples.

Table S1. RNA-seq read statistics.

Sample Barcode	Replicate Number	Group	Sampling Timepoint (h)	Total Reads	Reads Aligning to NCTC 11168	Reads aligning to NCTC 12673
**BC01**	1	Infected	0.5	4675121	3624270	497744
**BC02**	1	Infected	1	4158337	3481256	254092
**BC03**	1	Infected	2	6029137	5401531	369292
**BC04**	2	Infected	0.5	7329874	6525606	465763
**BC05**	2	Infected	1	6627184	5896267	403863
**BC06**	2	Infected	2	6446221	5803434	361905
**BC07**	2	Control	0.5	8711385	8649245	41
**BC08**	2	Control	1	6454979	6405427	330
**BC09**	2	Control	2	7161982	7111778	6
**BC10**	3	Infected	0.5	7173169	6580235	360334
**BC11**	3	Infected	1	6510986	6113184	236263
**BC12**	3	Infected	2	5850072	5354073	320383
**BC13**	3	Control	0.5	7241333	7183906	65
**BC14**	3	Control	1	8448990	8381159	27
**BC15**	3	Control	2	6872600	6819512	28

Table S2. Relative transcription of NCTC 12673 phage genes during *C. jejuni* NCTC 11168 infection.

Locus Tag	Annotation	% AA coverage[a] (Kropinski *et al.* 2011)	Relative transcription (log_{10}(RPKM))[b]
ncR001[d]	Novel non-coding RNA	N/A	5.81
gp074	Gp23 major capsid protein	43	5.23
gp089	Hypothetical protein	21.3	4.89
gp060	Gp18 tail sheath protein	0	4.66
gp068	Conserved hypothetical protein	7.6	4.40
gp075	Hypothetical protein	0	4.66
gp087	Conserved hypothetical protein	0	4.45
gp069	Hypothetical protein	7.3	4.65
gp112	Conserved hypothetical protein	0	4.40
gp153	Hypothetical protein	13.2	4.38
gp051	Gp32 ssDNA binding protein	0	4.41
gp079	Conserved hypothetical protein	4.7	4.49
gp036	Gp19 tail tube protein	6.9	4.36
gp038	Conserved hypothetical protein	0	4.48
gp076	Gp21 prohead core scaffold and protease	0	4.35
gp119	Hypothetical protein	16.8	4.12
gp117	Conserved hypothetical protein	2.5	4.37
gp111	Conserved hypothetical protein	0	3.94
gp001	Hypothetical protein	7.3	4.18
gp122	Gp23 major head protein II	25.3	4.24
gp080	Gp13 neck protein	6.6	4.18
gp088	Hypothetical protein	8.5	4.08
gp085	Phosphatidylserine decarboxylase	5.1	4.13
gp086	Co-chaperonin GroES	0	4.16
gp019	Hypothetical protein	22.7	4.13
gp037	Gp19 tail tube protein II	0	4.07
gp044	Amidinotransferase	20.8	4.14
gp108	Conserved hypothetical protein	18.3	4.08
gp123	Gp14 head completion protein	0	3.98
gp160	Hypothetical protein	0	3.19
gp138	Putative exonuclease	9.5	3.87
gp163	Ribonucleotide reductase, small subunit	0	3.57
gp116	Conserved hypothetical protein	2.7	3.78
gp043	Gp45 sliding clamp protein	0	3.88
gp115	Hypothetical protein	0	3.59
gp062	Conserved hypothetical protein	0	3.88
gp170	Hypothetical protein	0	3.70
gp077	Phage hypothetical protein	0	3.44
gp168	RecA	0	3.79
gp094	Conserved hypothetical protein	2.4	3.54
gp083	Gp19 tail tube protein III	7.8	3.81

gp171	Hypothetical protein	0	3.20
gp152	Gp4 head completion protein	0	3.68
gp035	Hypothetical protein	0	3.78
gp104	Conserved hypothetical protein	0	3.76
gp017	Hypothetical protein	0	3.79
gp078	Gp20 portal vertex protein	16.9	3.76
gp110	Conserved hypothetical protein	0	3.26
gp066	Conserved hypothetical protein	0	3.80
gp082	Hypothetical protein	0	3.25
gp026	Hypothetical protein	0	3.68
gp158	Hypothetical protein	0	3.80
gp048	Putative RNAse H	0	3.55
gp020	Conserved hypothetical protein	0	3.62
gp166	Hypothetical protein	0	3.63
gp041	Gp6 baseplate wedge subunit	0	3.55
gp098	Hypothetical protein	0	3.47
gp050	Gp62 clamp loader subunit	0	3.46
gp064	Hypothetical protein	0	3.71
gp154	Hypothetical protein	0	3.13
gp126	Gp15 tail sheath stabilizer and completion protein	0	3.57
gp049	Hypothetical protein	0	3.32
gp030	Gp5 baseplate hub subunit and tail lysozyme	0	3.43
gp061	Hypothetical protein	0	3.30
gp093	Hypothetical protein	16.2	3.44
gp159	Hypothetical protein	0	3.16
gp162	Ribonucleotide reductase, large subunit	0	3.54
gp099	Hypothetical protein	0	3.39
gp067	Hypothetical protein	0	3.58
gp155	RnlA; RNA ligase	0	3.15
gp165	Conserved hypothetical protein	0	3.41
gp063	Gp30 DNA ligase	0	3.49
gp102	Putative methyltransferase	0	3.45
gp148	Conserved hypothetical protein	0	3.35
gp105	Thymidine kinase	0	3.22
gp140	Hypothetical protein	0	3.46
gp006	Potential dUTP pyrophosphatase	0	3.11
gp149	Hypothetical protein	10.1	2.86
gp012	Conserved hypothetical protein	0	3.33
gp025	Hypothetical protein	0	3.36
gp142	Conserved hypothetical protein	0	3.32
gp109	Gp3 tail completion and sheath stabilizer protein	0	3.33
gp032	Probable Gp5.4 conserved hypothetical protein	0	3.46
gp007	Hypothetical protein	0	3.39

gp151	Hypothetical protein	0	3.22
gp016	Conserved hypothetical protein	0	3.32
gp156	Conserved hypothetical protein	0	2.94
gp114	Conserved hypothetical protein	0	3.42
gp027	Hypothetical protein	0	3.44
gp132	Hypothetical protein	0	3.18
gp169	Hypothetical protein	0	3.40
gp005	Hypothetical protein	0	3.33
gp003	Gp44 sliding clamp loader subunit (DNA polymerase accessory protein)	0	3.28
gp065	Hypothetical protein	0	3.31
gp107	Gp61 DNA primase subunit	0	3.08
gp084	Hef85 homing endonuclease	0	3.32
gp092	Putative repair and recombination protein	0	2.97
gp015	Gp39; topoisomerase II large subunit	0	3.27
gp042	Gp25 baseplate wedge subunit	0	3.27
gp028	Hypothetical protein	0	2.91
gp008	Potential NAD(FAD)-utilizing dehydrogenase	0	3.29
gp164	Hypothetical protein	0	3.26
gp141	Conserved hypothetical protein	0	3.05
gp103	Hypothetical protein	0	3.02
gp039	Hypothetical protein	0	3.34
gp029	Conserved hypothetical protein	0	3.20
gp118	Gp46 recombination endonuclease	0	3.05
gp139	Conserved hypothetical protein	0	3.11
gp045	Gp41 DNA primase-helicase subunit	0	3.20
gp033	Hef34 homing endonuclease	0	3.11
gp021	Hypothetical protein	0	3.13
gp127	Conserved hypothetical protein	0	3.06
gp135	Hypothetical protein	0	3.30
gp128	Hypothetical protein	0	2.95
gp134	Hypothetical protein	0	3.37
gp009	Hypothetical protein	0	3.19
gp024	Putative peptidase	0	3.14
gp040	Conserved hypothetical protein	0	3.14
gp056	Thymidylate synthetase	0	3.19
gp002	Hypothetical protein	17.9	3.08
gp047	Conserved hypothetical protein; flagellar glycan binding protein[c]	0	3.11
gp091	DNA helicase UvsW	0	3.08
gp146	Conserved hypothetical protein	0	2.70
gp143	Hypothetical protein	0	2.82
gp053	Gp2 DNA end protector protein	0	2.96
gp147	Conserved hypothetical protein	0	3.03
gp034	Hypothetical protein	0	2.63

gp133	Conserved hypothetical protein	0	2.75
gp144	Conserved hypothetical protein	0	2.75
gp070	Hef71 homing endonuclease	0	2.98
gp101	Gp43 DNA polymerase	0	2.95
gp096	Possible methylase	0	2.80
gp121	Gp55 sigma factor for T4 late transcription	0	2.76
gp095	Conserved hypothetical protein	6.5	2.79
gp120	Gp47 recombination endonuclease	0	2.93
gp161	Putative poly A polymerase	0	2.93
gp018	Hypothetical protein	0	2.55
gp023	Hypothetical protein	0	2.57
gp136	Conserved hypothetical protein	0	2.92
gp145	Hypothetical protein	0	2.75
gp052	Hef53 homing endonuclease	0	2.84
gp097	Putative RecB family exonuclease	0	2.62
gp073	Hef74 homing endonuclease	0	2.94
gp022	Hypothetical protein	0	2.73
gp129	Hypothetical protein	0	2.33
gp150	Hypothetical protein	0	2.52
gp004	Hypothetical protein	0	2.83
gp055	Conserved hypothetical protein	0	2.58
gp157	Hypothetical protein	0	2.70
gp014	Hef15 homing endonuclease	0	2.88
gp137	Hypothetical protein	0	2.64
gp100	Hypothetical protein	0	2.89
gp167	Hef168 homing endonuclease	0	2.64
gp010	Hypothetical protein	0	2.66
gp113	Conserved hypothetical protein	0	2.64
gp106	Hypothetical protein	0	2.67
gp011	Hypothetical protein	0	2.54
gp057	Hef58 homing endonuclease	0	2.70
gp131	Conserved hypothetical protein	0	2.54
gp046	Hypothetical protein	0	2.09
gp090	Putative DEAD/DEAH box helicase	0	2.45
gp072	Hef73 homing endonuclease	0	2.48
gp059	Hef60 homing endonuclease	0	2.59
gp031	Hypothetical protein	0	2.04
gp058	Hef59 homing endonuclease	0	2.33
gp130	Hypothetical protein	0	1.04

[a]Percent amino acid (AA) coverage following mass spectrometry of purified virions as determined by Kropinski *et al.* (2011); "0" indicates no peptides mapping to predicted AA sequence were detected

[b]RPKM (reads per kilobase million) values for each gene

[c]Annotation updated according to Javed *et al.* (2015)

[d]Non-coding RNA

Table S3. Number and percentage of *C. jejuni* NCTC 11168 genes differentially expressed (DE) at each time point post NCTC 12673 phage infection.

	30 min p.i.		60 min p.i.		120 min p.i.	
**Differentially expressed host genes[a]**	**Number**	**% of total**	**Number**	**% of total**	**Number**	**% of total**
**Upregulated**	61	3.7 %	32	1.9 %	212	12.7 %
**Downregulated**	1	0.06 %	8	0.5 %	78	4.7 %
**Total DE**	62	3.7 %	40	2.4 %	290	17.4 %

[a]Genes were considered DE if they underwent a fold change of >1.5 or <-1.5 with a false discovery rate (FDR)-adjusted p-value of <0.05.

CAAGGTTATGGTAAGCTTATTATGTTAATTTTCTCTTTACTTCTAAAGAGACCAT
AGCCTGAGTAATAAAATAGAACACATAAGGTTAATACGCAGACCTAAAATAA
GGTCCCTCCTTTCAATCTTCAGATACTCTTAACCTGGTGTTCTCTAAACAT

Figure S2. Nucleotide sequence of the novel 158-bp non-coding RNA (ncRNA) identified between *gp012* and *gp013* (nucleotides 7110-7267) on the phage genome.

Table S4. KEGG pathways for *C. jejuni* NCTC 11168 genes statistically significantly downregulated at 30, 60 and 120 min post NCTC 12673 phage infection.

**Significantly downregulated pathways[a]**	**Significance value (*p*-value)[b]**		
**KEGG pathway**	**30 min**	**60 min**	**120 min**
cje00010 Glycolysis /gluconeogenesis	0.02	<0.01	0.01
cje00020 Citrate cycle (TCA cycle)	0.00	<0.01	<0.01
cje00190 Oxidative phosphorylation			0.02
cje00250 Alanine, aspartate and glutamate metabolism	0.03	0.01	
cje00520 Amino sugar and nucleotide sugar metabolism	0.03		0.03
cje00620 Pyruvate metabolism	0.03	<0.01	0.01
cje00640 Propanoate metabolism	0.04	<0.01	0.01
cje00650 Butanoate metabolism	0.04	0.03	0.01
cje00680 Methane metabolism	0.04		
cje00970 Aminoacyl-tRNA biosynthesis		0.02	
cje01100 Metabolic pathways	<0.01	<0.01	<0.01
cje01110 Biosynthesis of secondary metabolites	0.02	<0.01	
cje01120 Microbial metabolism in diverse environments	<0.01	<0.01	<0.01
cje01130 Biosynthesis of antibiotics	<0.01	<0.01	0.01
cje01200 Carbon metabolism	<0.01	<0.01	<0.01
cje01230 Biosynthesis of amino acids		<0.01	
cje02020 Two-component system		0.02	0.03
cje02030 Bacterial chemotaxis		0.03	0.03
cje02040 Flagellar assembly		0.03	

[a]Differentially expressed host genes for each condition were subjected to gene set enrichment analysis (GSEA) on annotated Kyoto Encyclopedia of Genes and Genomes (KEGG) pathways using GAGE with an FDR cutoff of <0.1. KEGG pathways along with their significance values at each time point are listed in Table 5.

[b]Numerical values correspond to FDR-adjusted p-values (<0.05 was considered to be significant). A blank space indicates that the corresponding category was not statistically significantly downregulated.

Table S5. Differentially expressed *C. jejuni* NCTC 11168 genes upon NCTC 12673 phage infection according to COG categories.

**Gene and annotation according to COG category[a]**		**Fold change (p-value)[b]**		
**Gene**	**Annotation**	**30 min (*P*)**	**60 min (*P*)**	**120 min (*P*)**
**Amino acid transport and metabolism**				
argB	acetylglutamate kinase			3.3 (<0.01)
argC	N-acetyl-gamma-glutamyl-phosphate reductase			2.72 (<0.01)
argD	putative acetylornithine/succinyldiaminopimelate aminotransferase			3.18 (<0.01)
aroQ	3-dehydroquinate dehydratase			-1.72 (0.04)
aspB	aspartate aminotransferase			-2.04 (0.01)
cj0731	putative ABC transport system permease			3.08 (0.02)
cj0732	ABC transport system ATP-binding protein			3.29 (<0.01)
cj0919c	putative ABC-type amino-acid transporter permease protein			-1.63 (0.03)
cj0920c	putative ABC-type amino-acid transporter permease protein			-2.3 (<0.01)
cj0980	putative peptidase			-1.72 (0.03)
cj1111c	putative MarC family integral membrane protein			-1.97 (0.04)
cj1581c	putative peptide ABC-transport system ATP-binding protein	5.38 (<0.01)	4.4 (<0.01)	4.82 (<0.01)
cj1583c	putative peptide ABC-transport system permease protein	2.13 (<0.01)		4.82 (<0.01)
ilvD	dihydroxy-acid dehydratase		-1.82 (0.02)	4.82 (<0.01)
leuA	2-isopropylmalate synthase			2.62 (<0.01)
leuC	3-isopropylmalate dehydratase large subunit			2.37 (<0.01)
leuD	3-isopropylmalate dehydratase small subunit			2.43 (<0.01)
livF	branched-chain amino-acid ABC transport system ATP binding protein			1.89 (0.02)
livH	branched-chain amino-acid ABC transport system permease protein			1.69 (0.04)
livJ	branched-chain amino-acid ABC transport system periplasmic binding protein			1.47 (0.05)

metE	5-methyltetrahydropteroyltriglutamate--homocysteine methyltransferase			1.8 (0.01)
peb1A	aspartate/glutamate-binding ABC transporter protein			-2.51 (0.01)
proA	gamma-glutamyl phosphate reductase			-1.88 (0.02)
putP	putative sodium/proline symporter			-1.96 (0.02)
sdaA	L-serine dehydratase			-1.82 (0.03)
sdaC	amino acid transporter			-1.76 (<0.01)
thrB	putative homoserine kinase			1.92 (<0.01)
trpD	anthranilate synthase component II			1.86 (0.02)

Carbohydrate transport and metabolism

Gene	Annotation	30 min (P)	60 min (P)	120 min (P)
cj0035c	putative efflux protein	2.08 (0.04)		3.05 (<0.01)
cj1241	putative MFS (Major Facilitator Superfamily) transporter protein			2.64 (0.02)
cj1548c	putative NADP-dependent alcohol dehydrogenase		-1.7 (0.04)	2.64 (0.02)
dcuA	anaerobic C4-dicarboxylate transporter			2.64 (0.02)
kgtP	alpha-ketoglutarate permease			-2.26 (0.04)
pyk	pyruvate kinase			-1.81 (0.03)

Cell cycle control, cell division, chromosome partitioning

Gene	Annotation	30 min (P)	60 min (P)	120 min (P)
pbpC	penicillin-binding protein			1.68 (0.04)

Cell motility

Gene	Annotation	30 min (P)	60 min (P)	120 min (P)
cj1506c	putative MCP-type signal transduction protein			-1.53 (0.03)
fliR	flagellar biosynthetic protein			2.7 (<0.01)

Cell wall/membrane/envelope biogenesis

Gene	Annotation	30 min (P)	60 min (P)	120 min (P)
cadF	outer membrane fibronectin-binding protein			-1.52 (0.04)
cj0313	putative integral membrane protein			2.12 (0.02)
cj0505c	putative aminotransferase (DegT family)			2.11 (<0.01)
cj0646	putative lipoprotein			1.74 (<0.01)
cj0826	putative integral membrane protein	3.07 (<0.01)		3.46 (<0.01)
cj0958c	putative membrane protein			1.79 (<0.01)

lnt	putative apolipoprotein N-acyltransferase	2.37 (0.02)		2.71 (0.01)
cj1180c	putative ABC transporter ATP-binding protein			3.41 (<0.01)
cj1434c	putative sugar transferase		1.89 (0.02)	3.41 (<0.01)
cj1442c	putative sugar transferase		2.04 (0.03)	3.41 (<0.01)
cj1661	possible ABC transport system permease	2.54 (<0.01)		2.27 (0.03)
cj1662	putative integral membrane protein			2.82 (<0.01)
iamB	putative ABC transport system permease			1.7 (0.03)
lpxC	UDP-3-O-[3-hydroxymyristoyl] n-acetylglucosamine deacetylase			-1.76 (<0.01)
murD	UDP-N-acetylmuramoylalanine--D-glutamate ligase			1.69 (0.03)
tonB1	TonB transport protein	2.53 (0.03)		3 (0.01)
tonB2	putative TonB transport protein	3.92 (<0.01)	2.67 (0.02)	3.13 (0.01)
**Coenzyme transport and metabolism**				
**Gene**	**Annotation**	**30 min (P)**	**60 min (P)**	**120 min (P)**
bioB	putative biotin synthase			1.73 (0.04)
birA	putative biotin--[acetyl-CoA-carboxylase] synthetase			2.19 (0.03)
chuC	putative haemin uptake system ATP-binding protein			2.49 (0.05)
cj0230c	putative transferase protein			1.68 (0.03)
hemN	putative oxygen-independent coproporphyrinogen III oxidase	1.84 (<0.01)		2.43 (<0.01)
cj0541	polyprenyl synthetase		2.11 (0.02)	1.8 (0.02)
cj1420c	putative methyltransferase		-1.73 (0.03)	1.8 (0.02)
hemA	glutamyl-tRNA reductase			1.65 (0.02)
hemC	porphobilinogen deaminase			1.63 (0.03)
hemH	putative ferrochelatase			2.27 (<0.01)
cj0725c	molybdopterin biosynthesis protein			-1.87 (0.04)
ribD	riboflavin-specific deaminase/reductase			3.24 (<0.01)
**Defense mechanisms**				
**Gene**	**Annotation**	**30 min (P)**	**60 min (P)**	**120 min (P)**
cj0140	hypothetical protein Cj0140			1.75 (0.03)
cj0424	putative acidic periplasmic protein	9.95 (<0.01)	10.51 (<0.01)	12.75 (<0.01)
cj1174	putative efflux protein (multidrug resistance protein)			2.45 (0.03)
cj1298	putative N-acetyltransferase		2.5 (0.02)	2.45 (0.03)
**Energy production and conversion**				

Gene	Annotation	30 min (P)	60 min (P)	120 min (P)
ackA	acetate kinase			-2.42 (<0.01)
acnB	aconitate hydratase			-2.16 (0.03)
cj0037c	putative cytochrome C	2.51 (0.01)		-2.16 (0.03)
cj0073c	conserved hypothetical protein Cj0073c			-2.38 (0.01)
cj0074c	putative iron-sulfur protein			-2.32 (0.02)
cj0604	putative polyphosphate kinase			-2.72 (<0.01)
cj0833c	putative oxidoreductase			-1.86 (0.03)
cydA	cytochrome bd oxidase subunit I			2.33 (0.01)
cydB	cytochrome bd oxidase subunit II			2.54 (<0.01)
gltA	citrate synthase			-2.55 (0.01)
hydB	Ni/Fe-hydrogenase large subunit			-1.77 (0.05)
hydC	Ni/Fe-hydrogenase B-type cytochrome subunit			-1.95 (<0.01)
icd	isocitrate dehydrogenase		-1.87 (0.04)	-2.03 (0.04)
lctP	L-lactate permease			-2.28 (<0.01)
oorB	OORB subunit of 2-oxoglutarate:acceptor oxidoreductase			-1.87 (0.03)
pta	putative phosphate acetyltransferase			-2.25 (0.02)
putA	putative proline dehydrogenase/delta-1-pyrroline-5-carboxylate dehydrogenase			-2.22 (<0.01)
sucC	succinyl-coA synthetase beta chain			-2.09 (0.03)
sucD	succinyl-coA synthetase alpha chain			-1.9 (0.05)
icd	isocitrate dehydrogenase		-1.87 (0.04)	-1.9 (0.05)
**Function unknown**				
**Gene**	**Annotation**	**30 min (P)**	**60 min (P)**	**120 min (P)**
cj0014c	putative integral membrane protein			-1.69 (0.04)
cj0177	putative iron transport protein			3.84 (<0.01)
cj0189c	conserved hypothetical protein Cj0189c			1.7 (0.03)
cj0341c	putative integral membrane protein			2.18 (0.04)
cj0449c	conserved hypothetical protein Cj0449c			-2.43 (0.02)
cj0602c	MOSC-domain containing protein			-2.2 (0.03)
cj0728	putative periplasmic protein			3.37 (<0.01)
cj1384c	hypothetical protein Cj1384c			3.64 (<0.01)
cj1660	putative integral membrane protein			2.85 (<0.01)
**General function prediction only**				

Gene	Annotation	30 min (P)	60 min (P)	120 min (P)
cj0145	putative TAT (Twin-Arginine Translocation) pathway signal sequence domain protein	2.5 (0.02)		3.55 (<0.01)
cj0184c	putative serine/threonine protein phosphatase			2.37 (<0.01)
cj0504c	putative oxidoreductase			2.31 (0.01)
cj0605	putative amidohydrolase			-2.2 (<0.01)
cj0620	conserved hypothetical protein Cj0620		2.71 (0.02)	-2.2 (<0.01)
cj0711	hypothetical protein Cj0711		2.41 (0.02)	2.45 (0.03)
cj0730	putative ABC transport system permease			2.76 (0.03)
cj0937	putative integral membrane protein	7.19 (<0.01)	4.54 (<0.01)	10.14 (<0.01)
cj1028c	putative purine/pyrimidine phosphoribosyltransferase	2.36 (0.02)		2.7 (<0.01)
cj1225	conserved hypothetical protein Cj1225			-1.88 (<0.01)
cj1373	putative ntegral membrane protein			1.89 (0.02)
cj1500	putative integral membrane protein			4.26 (<0.01)
cj1164c	hypothetical protein Cj1164c	-2.13 (0.03)		4.26 (<0.01)

**Inorganic ion transport and metabolism**				
**Gene**	**Annotation**	**30 min (P)**	**60 min (P)**	**120 min (P)**
arsB	arsenical pump membrane protein			2.14 (0.03)
ceuB	enterochelin uptake permease			3.36 (<0.01)
cfrA	ferric enterobactin uptake receptor			2.33 (0.03)
cft	ferritin			-2.05 (0.02)
chuA	haemin uptake system outer membrane receptor	7.73 (<0.01)	4.9 (<0.01)	6.84 (<0.01)
chuD	putative haemin uptake system periplasmic haemin-binding protein	3.9 (<0.01)	2.73 (0.02)	3.31 (<0.01)
cj0167c	putative integral membrane protein	3.35 (<0.01)	2.71 (0.01)	5.35 (<0.01)
cj0178	putative TonB-denpendent outer membrane receptor	4.5 (<0.01)		4.86 (<0.01)
cj0186c	putative TerC family integral membrane protein			2.27 (<0.01)
cj0727	putative periplasmic solute-binding protein			2.5 (0.02)
cj0909	putative periplasmic protein			-2.73 (<0.01)
cj1609	putative sulfate adenylyltransferase			-1.7 (0.02)
cj1613c	putative pyridoxamine 5'-phosphate oxidase	3.34 (<0.01)	3.25 (<0.01)	2.47 (<0.01)
cj1658	putative iron permease	2.4 (0.01)		2.93 (<0.01)
feoB	ferrous iron transport protein			-1.64 (0.04)
katA	catalase	3.1 (<0.01)		3.42 (<0.01)
kdpB	potassium-transporting ATPase B chain			3.61 (<0.01)
ktrA	putative K+ uptake protein	4.08 (<0.01)	2.78 (<0.01)	6.99 (<0.01)

ktrB	putative K+ uptake protein	3.63 (<0.01)	2.4 (0.01)	5.8 (<0.01)
ppk	polyphosphate kinase			-1.9 (0.03)
pstS	putative periplasmic phosphate binding protein			1.8 (0.02)
chuB	putative haemin uptake system permease protein	3.02 (<0.01)		1.8 (0.02)
**Intracellular trafficking, secretion, and vesicular transport**				
**Gene**	**Annotation**	**30 min (P)**	**60 min (P)**	**120 min (P)**
cj0975	putative outer-membrane protein	2.77 (0.01)	2.43 (0.05)	3.13 (<0.01)
exbB1	biopolymer transport protein			3.76 (<0.01)
exbD3	putative ExbD/TolR family transport protein			1.84 (0.04)
ffh	signal recognition particle protein			1.64 (0.05)
secY	preprotein translocase subunit			2.49 (<0.01)
**Lipid transport and metabolism**				
**Gene**	**Annotation**	**30 min (P)**	**60 min (P)**	**120 min (P)**
acs	acetyl-coenzyme A synthetase		-2.53 (<0.01)	-2.03 (0.02)
cj0807	putative oxidoreductase			-1.69 (0.03)
fabH	3-oxoacyl-[acyl-carrier-protein] synthase			1.56 (0.02)
p19	periplasmic protein p19			2.23 (0.01)
plsC	putative 1-acyl-SN-glycerol-3-phosphate acyltransferase			1.77 (0.02)
pycA	pyruvate carboxylase A subunit			-2.04 (<0.01)
**Nucleotide transport and metabolism**				
**Gene**	**Annotation**	**30 min (P)**	**60 min (P)**	**120 min (P)**
nrdA	ribonucleoside-diphosphate reductase alpha chain			1.93 (0.01)
oorD	OORD subunit of 2-oxoglutarate:acceptor oxidoreductase			-1.76 (0.04)
purB	adenylosuccinate lyase			2.15 (0.02)
purH	phosphoribosylaminoimidazolecarboxamide formyltransferase / IMP cyclohydrolase			2.64 (<0.01)
purL	phosphoribosylformylglycinamidine synthase			2.2 (<0.01)
purE	phosphoribosylaminoimidazole carboxylase catalytic subunit		-1.95 (<0.01)	2.2 (<0.01)
**Posttranslational modification, protein turnover, chaperones**				
**Gene**	**Annotation**	**30 min (P)**	**60 min (P)**	**120 min (P)**
cj0190c	conserved hypothetical protein Cj0190c			1.95 (<0.01)

cj0911	putative periplasmic protein			-2.17 (0.02)
cj0954c	putative DnaJ-like protein			2.31 (0.01)
cj1501	conserved hypothetical protein Cj1501			4.14 (<0.01)
dnaK	heat shock protein DnaK			1.59 (0.05)
dsbD	putative thiol:disulphide interchange protein			-2.1 (0.03)
htpG	hsp90 family heat shock protein			1.58 (0.04)
**Replication, recombination and repair**				
**Gene**	**Annotation**	**30 min (P)**	**60 min (P)**	**120 min (P)**
cj0208	putative DNA modification methylase (adenine-specific methyltransferase)			2.93 (<0.01)
cj0777	putative ATP-dependent DNA helicase			1.88 (0.02)
cj1254	hypothetical protein Cj1254			2.63 (<0.01)
ruvC	crossover junction endodeoxyribonuclease			3.51 (<0.01)
ssb	single-strand DNA binding protein			2.5 (0.01)
topA	DNA topoisomerase I			1.53 (0.04)
xseA	exodeoxyribonuclease VII large subunit			1.85 (0.03)
**Secondary metabolites biosynthesis, transport and catabolism**				
**Gene**		**30 min (P)**	**60 min (P)**	**120 min (P)**
cj0021c	putative fumarylacetoacetate (FAA) hydrolase family protein			-2.26 (0.02)
cj1199	putative iron/ascorbate-dependent oxidoreductase	2.31 (0.03)		-2.26 (0.02)
**Signal transduction mechanisms**				
**Gene**	**Annotation**	**30 min (P)**	**60 min (P)**	**120 min (P)**
cj0241c	putative iron-binding protein	2.69 (<0.01)		3.54 (<0.01)
cj1224	putative iron-binding protein			-1.98 (0.02)
cj1258	putative phosphotyrosine protein phosphatase			-2.53 (<0.01)
cstA	putative integral membrane protein (CstA homolog)		-1.82 (0.03)	-2.53 (<0.01)
typA	GTP-binding protein TypA homolog			-2.53 (<0.01)
**Transcription**				
**Gene**	**Annotation**	**30 min (P)**	**60 min (P)**	**120 min (P)**
cj1556	putative transcriptional regulator			1.83 (0.05)
hrcA	putative heat shock regulator			2.01 (<0.01)
rpoA	DNA-directed RNA polymerase alpha chain			1.87 (<0.01)

rpoC	DNA-directed RNA polymerase beta chain			1.55 (0.05)
**Translation, ribosomal structure and biogenesis**				
**Gene**	**Annotation**	**30 min (P)**	**60 min (P)**	**120 min (P)**
cj0123c	putative tRNA-dihydrouridine synthase			2.24 (<0.01)
cj0154c	putative tetrapyrrole methylase family protein			2.18 (0.01)
cj0225	putative acetyltransferase			3.27 (<0.01)
rplY	putative 50S ribosomal protein L25 (general stress protein Ctc)			1.64 (0.04)
cj0495	putative methyltransferase domain protein			2.86 (<0.01)
cj0636	NOL1/NOP2/sun family protein			2.14 (0.02)
cj1172c	conserved hypothetical protein Cj1172c			1.63 (0.05)
cj1709c	putative ribosomal pseudouridine synthase			1.85 (0.03)
cj1713	putative radical SAM domain protein			2.28 (0.02)
fmt	methionyl-tRNA formyltransferase			2.16 (<0.01)
prfA	peptide chain release factor 1			2.65 (<0.01)
pth	peptidyl-tRNA hydrolase			1.77 (0.02)
rimM	putative 16S rRNA processing protein			2.02 (<0.01)
rplD	50S ribosomal protein L4			1.59 (0.03)
rplF	50S ribosomal protein L6			1.91 (<0.01)
rplJ	50S ribosomal protein L10			1.94 (<0.01)
rplK	50S ribosomal protein L11			1.85 (0.03)
rplM	50S ribosomal protein L13			1.8 (0.01)
rplT	50S ribosomal protein L20			1.7 (<0.01)
rplW	50S ribosomal protein L23			2.17 (<0.01)
rpmC	50S ribosomal protein L29			1.77 (0.04)
rpmE	50S ribosomal protein L31			2.51 (<0.01)
rpsB	30S ribosomal protein S2			1.87 (<0.01)
rpsC	30S ribosomal protein S3			1.57 (0.02)
rpsD	30S ribosomal protein S4			1.71 (0.02)
rpsE	30S ribosomal protein S5			1.69 (0.02)
rpsF	30S ribosomal protein S6			1.63 (0.03)
rpsH	30S ribosomal protein S8			1.86 (0.01)
rpsI	30S ribosomal protein S9			2.46 (<0.01)
rpsK	30S ribosomal protein S11			2.31 (<0.01)
rpsM	30S ribosomal protein S13			1.57 (0.05)

rpsQ	30S ribosomal protein S17			2.05 (0.02)
rpsR	30S ribosomal protein S18			2.12 (<0.01)
rpsS	30S ribosomal protein S19			1.73 (0.04)
rpsT	30S ribosomal protein S20			1.74 (0.04)
serS	seryl-tRNA synthetase			-1.64 (0.04)
tlyA	putative haemolysin			2.37 (<0.01)
trmD	tRNA (guanine-N1)-methyltransferase		2.22 (0.03)	2.68 (<0.01)
tsf	elongation factor TS			2.04 (<0.01)
**Uncategorized**				
**Gene**	**Annotation**	**30 min (P)**	**60 min (P)**	**120 min (P)**
bioC	putative biotin synthesis protein	2.39 (0.04)		2.95 (0.02)
cca	putative multifunctional Cca protein			2.13 (<0.01)
cdtA	cytolethal distending toxin A			-1.76 (0.03)
cgb	single domain haemoglobin			2.56 (0.02)
cj0011c	putative non-specific DNA binding protein			-2.12 (<0.01)
cj0030	hypothetical protein Cj0030	2.13 (0.04)	2.41 (<0.01)	2.49 (<0.01)
cj0038c	putative poly(A) polymerase family protein			3.33 (<0.01)
cj0069	hypothetical protein Cj0069			-2.03 (0.04)
cj0075c	putative oxidoreductase iron-sulfur subunit			-2.52 (<0.01)
cj0080	putative membrane protein			3.14 (0.01)
cj0093	putative periplasmic protein			1.64 (0.02)
cj0111	putative periplasmic protein			1.98 (<0.01)
cj0120	hypothetical protein Cj0120			2.19 (0.03)
cj0124c	putative membrane protein			2.2 (<0.01)
cj0223	pseudogene (putative IgA protease family protein)			1.72 (0.05)
cj0254	hypothetical protein Cj0254			-1.54 (0.04)
cj0260c	small hydrophobic protein		2.13 (0.02)	2.56 (0.02)
cj0267c	putative integral membrane protein			2.53 (0.02)
cj0295	putative acetyltransferase			2.4 (0.02)
cj0323	hypothetical protein Cj0323			1.82 (0.05)
cj0344	hypothetical protein Cj0344			-2.17 (0.01)
cj0350	hypothetical protein Cj0350			-1.77 (0.02)
cj0391c	hypothetical protein Cj0391c	1.64 (0.03)		-1.77 (0.02)
cj0406c	putative lipoprotein			-1.66 (0.05)
cj0414	putative oxidoreductase subunit			2.15 (0.04)

cj0421c	putative integral membrane protein	2.59 (0.03)		6.38 (<0.01)
cj0423	putative integral membrane protein	5.73 (<0.01)	3.36 (<0.01)	16.14 (<0.01)
cj0425	putative periplasmic protein	9.01 (<0.01)	9.1 (<0.01)	10.39 (<0.01)
cj0427	hypothetical protein Cj0427			-2.11 (0.02)
cj0444	pseudogene (putative TonB-denpendent outer membrane receptor)	2.04 (0.04)	2.12 (0.04)	2.29 (<0.01)
cj0448c	putative MCP-type signal transduction protein			-2.17 (0.05)
cj0494	putative exporting protein			2.57 (<0.01)
cj0515	putative periplasmic protein			1.82 (0.02)
cj0520	putative membrane protein			2.2 (<0.01)
cj0522	putative Na+/Pi cotransporter protein			2.68 (<0.01)
cj0544	putative integral membrane protein			2.3 (<0.01)
cj0566	hypothetical protein Cj0566	7.23 (<0.01)	3.99 (<0.01)	2.3 (<0.01)
cj0584	hypothetical protein Cj0584			1.94 (0.03)
cj0590	putative SAM-dependent methyltransferase			1.86 (0.03)
cj0648	putative membrane protein			2.22 (0.01)
cj0651	putative integral membrane protein		2.8 (<0.01)	2.22 (0.01)
cj0694	putative periplasmic protein			1.57 (0.03)
cj0700	hypothetical protein Cj0700			-1.82 (0.04)
cj0729	putative type I phosphodiesterase/nucleotide	2.77 (0.02)	2.57 (0.02)	4.25 (<0.01)
cj0735	putative periplasmic protein	2.58 (<0.01)		4.25 (<0.01)
cj0736	hypothetical protein Cj0736	5.35 (<0.01)	2.49 (0.04)	4.25 (<0.01)
cj0742	pseudogene (putative outer membrane protein)	4.71 (<0.01)	4.07 (<0.01)	3.91 (<0.01)
cj0752	pseudogene (IS element transposase)	2.72 (0.02)	2.68 (0.02)	3.54 (<0.01)
cj0794	hypothetical protein Cj0794	3.22 (<0.01)		2.6 (0.02)
cj0834c	ankyrin repeat-containing putative periplasmic protein			-1.82 (0.04)
cj0873c	hypothetical protein Cj0873c			-2.14 (0.01)
cj0910	putative periplasmic protein			-2.21 (<0.01)
cj0944c	putative periplasmic protein		-1.88 (0.03)	-2.21 (<0.01)
cj0951c	putative MCP-domain signal transduction protein			-1.8 (0.04)
cj0967	putative periplasmic protein	4.42 (<0.01)		3.8 (<0.01)
cj1012c	putative membrane protein			2.3 (0.01)
cj1041c	putative periplasmic ATP/GTP-binding protein	2.68 (<0.01)		1.95 (0.04)
cj1078	putative periplasmic protein	2.49 (0.05)		1.95 (0.04)
cj1159c	small hydrophobic protein	2.62 (0.03)		2.65 (0.04)
cj1162c	putative heavy-metal-associated domain protein	3.35 (<0.01)		3.31 (<0.01)
cj1169c	putative periplasmic protein			2.21 (0.02)

cj1307	putative amino acid activating enzyme			-1.67 (0.02)
cj1322	hypothetical protein Cj1322	3.48 (<0.01)		2.42 (0.05)
cj1383c	hypothetical protein Cj1383c	2.39 (0.04)		2.42 (0.05)
cj1389	pseudogene (putative C4-dicarboxylate anaerobic carrier)	2.11 (<0.01)		2.42 (0.05)
cj1395	pseudogene (putative MmgE/PrpD family protein)	2.82 (0.02)		2.42 (0.05)
cj1422c	putative sugar transferase			2.42 (0.05)
cj1459	hypothetical protein Cj1459			2.42 (0.05)
cj1664	putative periplasmic thiredoxin	2.66 (<0.01)		2.42 (0.05)
cj1665	putative lipoprotein thiredoxin			2.42 (0.05)
cj1677	putative lipoprotein	5.48 (<0.01)	2.99 (<0.01)	2.42 (0.05)
cj1712	hypothetical protein Cj1712			2.42 (0.05)
cetB	bipartate energy taxis response protein cetB	2.21 (0.03)		2.42 (0.05)
cmeA	periplasmic fusion protein CmeA (multidrug efflux system CmeABC)	2.6 (<0.01)		1.99 (<0.01)
cmeB	inner membrane efflux transporter CmeB (multidrug efflux system CmeABC)	2.09 (<0.01)		2.14 (<0.01)
cmeC	outer membrane channel protein CmeC (multidrug efflux system CmeABC)	2.04 (<0.01)		2.02 (<0.01)
ctsP	putative ATP/GTP-binding protein	2.8 (<0.01)	2.55 (<0.01)	2.47 (0.01)
dba	disulphide bond formation protein	2.44 (0.04)		3.32 (<0.01)
dccR	two-component regulator			-1.93 (0.02)
dprA	DNA processing protein A			1.91 (0.02)
dsbI	disulphide bond formation protein	2.33 (0.03)		4.15 (<0.01)
exoA	exodeoxyribonuclease			1.97 (0.03)
fdxA	ferredoxin			-1.89 (0.05)
flaC	flagellin			-2.1 (0.03)
glpT	pseudogene (putative glycerol-3-phosphate transporter)			-2.12 (0.05)
hldD	ADP-glyceromanno-heptose 6-epimerase			-1.57 (0.03)
kdpA	pseudogene (potassium-transporting ATPase A chain)	2.76 (<0.01)	2.62 (<0.01)	2.27 (<0.01)
kpsC	capsule polysaccharide modification protein			1.6 (0.04)
lolA	putative outer-membrane lipoprotein carrier protein precursor			-1.89 (0.03)
mreC	homolog of *E. coli* rod shape-determining protein			1.72 (0.04)
nrdB	ribonucleoside-diphosphate reductase beta chain			1.66 (0.04)
cj0096	GTP-binding protein			2.25 (<0.01)
omp50	50 kDa outer membrane protein precursor	2.43 (0.05)		3.89 (<0.01)
pglA	GalNAc transferase			1.99 (0.04)

pseI	Pse synthetase	-1.67 (0.03)
rpmJ	50S ribosomal protein L36	1.73 (0.04)
rpoB	DNA-directed RNA polymerase beta chain	1.96 (0.01)
trmE	putative tRNA modification GTPase	2.16 (<0.01)
ubiD	putative 3-octaprenyl-4-hydroxybenzoate carboxy-lyase	1.61 (0.03)

[a]Host genes found to be differentially expressed between infected and control samples for at least one time point were assigned to one of 25 functional categories based on clusters of orthologous groups (COG) classification. Genes were assigned according to the COG function list for *C. jejuni jejuni* NCTC 11168 according to the Integrated Microbial Genomes and Microbiomes (IMG/M) database (https://img.jgi.doe.gov). Gene annotations were obtained from NCBI. [b]For each gene, fold change in expression post NCTC 12673 phage infection is listed followed by the adjusted *P*-value (*P*) in brackets. Fold change values for genes whose transcripts were not significantly differentially expressed are not shown.

A

```
Cj0423    1 MEGASGGSIFVT----ILLFILYMLPAIIGLSRKHSNWLIISLLNLFFGWTIIVWLVCLI
Imm       1 METLVAGSIFMVLVSGVLAIIIYMLPWFIALMRGSKSTVGIFFASLLFNWSIIGWFITFI

Cj0423   57 WSFTGSFK------QTIIIKEKNEK
Imm      61 WSIAGETKKSAQPNQVIIIREKE--
```

B

```
Cj0424    1 MTKFLSICSLIAM-------L-------LSGCGSDFPGQPSDVARVQQNKYPNGNLKKEI
YwqK      1 MENEYDMKSIKEKGVDFEDLWFSSVSDEILDNPEDENGQPFT--GLAYELYPNGQIIYFT

Cj0424   47 PYNKDSRIHGLKRAFYDNGQLRAEENYKNGKKDGISREYSRNGQLLEEVHFKDNRGYGDF
YwqK     59 KY-KNGLAHGLTCEFYENGNKKSEKEYRYGQLHGISIIWFENGRKKSEQQYEHSILI---

Cj0424  107 ASYYENGNMRAKGKLLGYNEDGMPEFEGNYKEYYENGTLMCDYNFDNK-GKFDGVQKRYD
YwqK    115 ----------------------------SEKNWDEEGNLLNKYEIDTDSPHFEILESRRE

Cj0424  166 ENGALEDEENYKNGLKNGVFREYKKGEIVREEEYKNGILVAKPKN
YwqK    147 THINLGRE-------------------------------------
```

Figure S3. BLASTp alignment between *C. jejuni* NCTC 11168 protein Cj0423 and T4 phage protein Imm (**A**) and between *C. jejuni* NCTC 11168 protein Cj0424 and *Bacillus subtilis* strain 168 antitoxin protein YwqK (**B**). Alignments were generated using T-Coffee [2] and Boxshade (http://sourceforge.net/projects/boxshade/). Black shading indicates the consensus sequence, grey shading indicates similar residues.
